# Impact of including two types of destoned olive cakes in pigs’ diets on fecal bacterial composition and study of the relationship between fecal microbiota, feed efficiency, gut fermentation, and gaseous emissions

**DOI:** 10.3389/fmicb.2024.1359670

**Published:** 2024-06-12

**Authors:** Dhekra Belloumi, Paloma García-Rebollar, Salvador Calvet, M. Pilar Francino, Mariana Reyes-Prieto, Jorge González-Garrido, Laia Piquer, Ana Isabel Jiménez-Belenguer, Almudena Bermejo, Carmen Cano, Alba Cerisuelo

**Affiliations:** ^1^Centro de Investigación y Tecnología Animal, Instituto Valenciano de Investigaciones Agrarias, Segorbe, Spain; ^2^Institute for Animal Science and Technology, Universitat Politècnica de València, Valencia, Spain; ^3^Departamento de Producción Agraria, ETSIAAB, Universidad Politécnica de Madrid, Madrid, Spain; ^4^FISABIO-Public Health, Foundation for the Promotion of Health and Biomedical Research in the Valencian Region, Valencia, Spain; ^5^CIBER en Epidemiología y Salud Pública, Madrid, Spain; ^6^Sequencing and Bioinformatics Service, Foundation for the Promotion of Health and Biomedical Research in the Valencian Region, Valencia, Spain; ^7^rDepartamento de Biotecnología, Universitat Politècnica de València, Valencia, Spain; ^8^Centro de Citricultura y Producción Vegetal, Instituto Valenciano de Investigaciones Agrarias, Moncada, Spain

**Keywords:** olive cake, pig, fecal bacterial composition, efficiency, gaseous emissions

## Abstract

The microbial population in the pig’s gastrointestinal tract can be influenced by incorporating fibrous by-products into the diets. This study investigated the impact of including two types of dried olive cake (OC) in pigs’ diets on fecal bacterial composition. The correlation between fecal microbiota and growth performance, nutrient digestibility, gut fermentation pattern and slurry gas emissions was also evaluated. Thirty male Pietrain x (Landrace x Large white) pigs (47.9 ± 4.21 kg) were assigned to three groups: a control group (C), a group fed a diet with 20% partially defatted OC (20PDOC), and a group fed a diet with 20% cyclone OC (20COC) for 21 days. Fecal samples collected before and after providing the experimental diets were analyzed for the V3-V4 region of the 16S rRNA gene. Pigs were weighed, and feed intake was recorded throughout the study. Potential ammonia and methane emissions from slurry were measured. No significant differences in alpha diversity indexes were found. The taxonomic analysis revealed that Firmicutes and Bacteroidota phyla were dominant at the phylum level across all groups. Differential abundance analysis using ALDEx showed significant differences among groups for various bacteria at the phylum, genus, and species levels at the end of the experiment. Pigs from 20PDOC and 20COC groups exhibited increased abundances of health-promoting bacteria, such as Plactomycetota at the phylum level and Allisonella and an unidentified genus from the Eggerthellaceae family at the genus level. These changes influenced short-chain fatty acids’ (SCFA) concentration in slurries, leading to greater acetic, butyric, caproic and heptanoic acids in OC-fed groups, especially 20COC pigs. A volatility analysis revealed significant positive correlations (*p* < 0.05) between Uncultured_Bacteroidales and Unculured_Selenomonadaceae and energy digestibility. Monoglobus and Desulfovibrio showed a positive significant (*p* < 0.05) correlation with total SCFA, indicating a high impact on gut fermentation. However, growth performance parameters and potential gas emission displayed no significant correlations with a specific bacterial genus. In conclusion, our results suggest that OC inclusion into pig diets could positively modulate and contribute to the gut microbiota’s favorable composition and functionality. Also, nutrient digestibility and gut fermentation patterns can be associated with specific microbial populations.

## 1 Introduction

Olive by-products, such as olive pomace, olive cake (OC), and olive leaves, can serve as a sustainable alternative source of nutrients for animal feeding. The use of these by-products in animal nutrition reduces livestock feed’s environmental and economic impact (Makkar and Ankers, 2014; Salemdeeb et al., 2017) and lessens waste in the industry (Molina-Alcaide and Yáñez-Ruiz, 2008). Understanding its nutritional and nutraceutical potential is essential to determine its impact on animal performance and health. OC comprises olive pulp and skin (Dermeche et al., 2013), and contains a high amount of dietary fiber (around 55% of neutral detergent fiber, NDF, and 40% of acid detergent fiber, ADF). Its fiber is characterized by a high proportion of lignin (17%) (De Blas et al., 2019). Its fat content is variable, ranging from 0 to 16.6% (De Blas et al., 2015). However, OC’s composition and nutritional value can be highly influenced by its origin, industrial processing, or subsequent conditioning (De Blas et al., 2015; Ferrer et al., 2020). Additionally, OC has been identified as a valuable source of phenolic compounds (up to 0.3%), including hydroxytyrosol and its derivatives, secoiridoids, flavones (luteolin and apigenin), and elenolic acid and its derivatives (López-Salas et al., 2021). These compounds have been found to have antioxidant and antimicrobial properties that could be highly beneficial for promoting a healthy gut microbiota and overall gut health.

Traditionally, fibrous by-products such as OC have been cautiously integrated into pig diets with limited inclusion rates due to the negative impact of fiber on nutrient digestibility and energy utilization (Ferrer et al., 2018). However, precisely due to its high amount of fiber and bioactive compounds, the inclusion of OC in diets can be associated with changes in gut microbiota patterns and health. It is known that both the quantity and source of dietary fiber, significantly influence the composition of gut microbiota in pig fecal samples and the quantity and profile of short-chain fatty acids (SCFA) produced by the microbiota (Zhao et al., 2019). Zhao et al. (2020) reported, in this regard, that SCFA concentration in feces correlated positively with the fermentability and digestibility of insoluble dietary fibers. Also, it is known that SCFAs can provide up to 25% of the pigs’ total available energy in the hindgut (Pu et al., 2020) and improve the host’s intestinal integrity and immune system (Chambers et al., 2015). Furthermore, recent studies have demonstrated that SCFA could improve various health parameters in humans and mice through a shift in gut bacterial composition (Peterson et al., 2022; Tang et al., 2023). Studies on the effects of OC on pigs’ gut microbiota and fermentation patterns are still scarce in the literature, with only a few recent research indicating that OC might modify fecal microbiota composition in cows (Russo et al., 2023) and sows (Sánchez et al., 2022). On the other hand, several studies have investigated the effects of the bioactive compounds derived from olives in animal feeds on gut microbial composition. One such compound, apigenin, has been found to inhibit the growth of *Bacteroides galacturonicus* and *Enterococcus caccae*, while also promoting microbial diversity and the production of SCFA in human fecal colonic batch culture (Wang et al., 2017). In a study conducted by Martínez et al. (2019), it was observed that feeding laboratory mice with virgin olive oil resulted in the reduction of undesirable bacteria of the Desulfovibrionaceae, Spiroplasmataceae, and Helicobacteraceae families and the increase in the abundance of Erysipelotrichaceae and Sutterellaceae families in their gut, indicating a possible interplay between olive oil phenolic compounds and microbial composition. However, Liehr et al. (2017) reported no significant differences in the gut microbiota composition and function of pigs challenged with *E. coli*-derived lipopolysaccharides injection fed with olive oil bioactive compounds, although they observed positive effects on gut integrity. Then, while feeding olive by-products into the livestock diet is considered a sustainable practice, its impact on gut microbiota composition, fermentation patterns and, therefore, gut health in growing pigs, has yet to be established.

Additionally, the gut microbiota is a complex and dynamic ecosystem that plays key roles in maintaining nutritional, physiological, and metabolic functions in swine (Fouhse et al., 2016), contributing to pig health and sustainability. Recent insight indicates that gut microbes are important in regulating pig growth (Sato et al., 2019; Xin et al., 2020). Indeed, the gut bacterial population is intricately linked to nutrient utilization, with a diverse microbial community positively impacting nutrient digestibility, thereby enhancing overall feed efficiency (Le Sciellour et al., 2018). However, there is no consensus on the main microbial populations involved in these effects. Also, derived from the close relationship between dietary nutrient utilization, excreta composition, and gas emission (Beccaccia et al., 2015; Ferrer et al., 2018), it is possible that emissions from slurry could also be linked to certain microbial populations involved in feed efficiency. In this regard, Wang et al. (2021) have shown that different types of manure have varying effects on gas emissions during composting due to differences in their characteristics and microorganism communities. However, studies on this issue are still scarce in the literature. Thus, the control of feed efficiency, nutrient digestion, and gas emissions in pigs is critical for maintaining animal health and pig sustainability, and its relation with gut microbiota is still not fully understood.

The objectives of the present study were to investigate the effects on the fecal bacterial composition of including two dried OC in pigs’ diet that were obtained at different steps of the drying process, a partially defatted olive cake (PDOC) and a cyclone olive cake (COC), and to assess the correlation between gut microbial communities and growth performance, energy and protein digestibility and slurry gas emission.

## 2 Materials and methods

### 2.1 Animals, diets, and experimental design

Thirty male Pietrain x (Landrace x Large white) growing pigs of 47.9 ± 4.21 kg body weight (BW) were used in this experiment. Pigs were distributed in two batches of 15 animals each and kept in individual pens with free access to water and feed throughout the experiment. On day 1 of the experimental period, pigs were randomly divided into three dietary treatment groups (5 animals/batch and dietary treatment). The weight of these animals was considered in this distribution, so the mean BW was similar among treatment groups in each batch. These experimental treatments consisted of a control feed (C), a feed with 20% of PDOC (20PDOC), and a feed with 20% of COC (20COC). These feeds were provided to the animals for 21 consecutive days. The C feed contained corn, wheat, and soybean meal and reached the nutrient requirements reported by FEDNA for growing pigs (De Blas et al., 2013). The other two experimental treatments were obtained by substituting 20% of the energetic part of the C feed with PDOC and COC in 20PDOC and 20COC diets, respectively. The two types of olive cake (PDOC and COC) were provided by Valoriza Energy, a company in the Sacyr-Vallehermoso group devoted to pomace drying and olive pomace oil extraction. The PDOC was obtained after partially removing pits and drying fresh OC with a trommel dryer, using recovery heat gasses from a gas turbine installed in a cogeneration plant. During this process, the residual lighter pomace particles accumulated on the air decanters of the trommel and were separately recollected and named COC. The chemical composition of PDOC and COC is shown in Table 1. The ingredients and chemical composition of the experimental diets are shown in Table 2. After 14 days of adaptation to diets, 24 of the 30 animals included in the experiment (8 animals/treatment) were subjected to a 4-day nutrient balance (balance period) followed by a 3-day balance (emissions period) in which urine and feces were collected separately and individually (per animal) using metabolism pens, as reported in Ferrer et al. (2021). The rest of the animals were maintained in their original pens. According to pigs’ needs, the barn temperature was maintained at 20–25°C during the experiment.

**Table 1:** Analyzed chemical composition of partially defatted olive cake and cyclone olive cake (g/kg DM, unless otherwise specified).

Analyzed chemical composition	PDOC	COC
Dry matter	919	942
Ash	67.6	77.7
Crude protein	82.4	102
Crude fat	116	168
Total dietary fiber	561	467
NDF^a^	561	467
ADF^b^	327	283
ADL^c^	187	166
Insoluble fiber^d^	506	392
Soluble fiber^e^	55.3	75.4
Gross energy, MJ/kg	22.5	23.9
**Phenolic compounds, mg/g DM**		
Hydroxytyrosol	12.17	13.67
Tyrosol	2.30	2.55
Verbascoside	0.70	0.82
Oleacein	1.80	2.15
p-coumaric acid	0.15	0.17

PDOC = partially defatted olive cake; COC = cyclone olive cake.

a Neutral detergent fiber.

b Acid detergent fiber.

c Acid detergent lignin.

d Calculated as the NDF corrected by the protein content in the residue (Beccaccia et al., 2015).

e Calculated as the difference between total dietary fiber and NDF corrected by the protein content in the residue (Beccaccia et al., 2015).

**Table 2:** Ingredients and chemical compositions of the experimental diets (g/kg DM, unless otherwise specified).

	Treatments		
	C	20PDOC	20COC
**Ingredients**			
Corn	620	493	493
Wheat	100	79.4	79.4
Soybean meal (47.0% crude protein)	251	199	199
Partially defatted olive cake	–	200	–
Cyclone olive cake	–	–	200
Calcium carbonate	10.5	10.5	10.5
Calcium phosphate	9.4	9.4	9.4
Sodium chloride	4.3	4.3	4.3
DL-methionine	0.32	0.25	0.25
L-lysine HCL	1.46	1.16	1.16
Premix^a^	3.0	3.0	3.0
**Analyzed nutrients**			
Dry matter	885	890	894
Ash	54.2	61.2	62.1
Crude protein	209	187	185
Crude fat	38.6	65.2	80.6
Total dietary fiber	105	191	164
Gross energy, MJ/kg	18.3	19.0	19.3
**Calculated nutrients** ^b^			
Metabolic energy, MJ/kg	15.5	13.6	14.0
Calcium	0.77	0.96	0.96
Phosphorus	0.65	0.60	0.60
**Phenolic compounds, mg/g DM**			
Hydroxytyrosol	ND^c^	2.31	2.62
Tyrosol	ND^c^	0.41	0.45
Verbascoside	ND^c^	0.14	0.18
Oleacein	ND^c^	0.36	0.40
p-coumaric acid	ND^c^	0.09	0.09

C = control diet; 20PDOC = experimental diet with 20% of partially defatted olive cake in replacement of the energetic part of the control diet; 20COC = experimental diet with 20% of cyclone olive cake in replacement of the energetic part of the control diet.

a Vitamin and mineral premix contain per kg premix: 50 mg/kg biotin, 3,333,333 IU/kg of vitamin A (retinyl acetate); 666,667 IU/kg of vitamin D3 (cholecalciferol); 33,333 mg/kg of Vitamin E (tot-rac-α-tocopheryl acetate); 667 mg/kg of Vitamin K3 (menadian); 1,250 mg/kg of Vitamin B1, 2,400 mg/kg of Vitamin B2, 1,750 mg/kg of Vitamin B6; 10 mg/kg of Vitamin B12; 17,000 mg/kg of nicotinic acid; 10,000 mg/kg of calcium pantothenate; 600 mg/kg of folic acid; 100,000 mg/kg of choline chloride; 40,000 mg/kg of zinc dioxide (32,132 mg /kg of zinc); 23,400 mg/kg of iron sulfate monohydrated (7,681.4 mg/kg of iron); 2,000 mg/kg of cupric sulfate pentahydrate (508.6 mg/kg of copper); 250 mg/kg potassium iodide (191.25 mg/kg of iodine); 15,000 mg/kg manganese sulfate monohydrated (4,876.5 mg/kg of manganese); 100 mg/kg sodium selenite (45.62 mg/kg of selenium).

b Based on FEDNA feed tables (de Blas et al., 2019).

c Not-detectable.

### 2.2 Fecal samples for bacterial composition analysis

Fecal samples were obtained from all animals before (time 0) and after 21 days (time 1) of providing the experimental feeds to perform the microbiota analyses. Feces were collected aseptically directly from the rectum by rectal stimulation, frozen immediately with liquid nitrogen (N), and stored at −80°C until analyses.

### 2.3 Fecal bacteria characterization by 16S rRNA gene amplicon sequencing and bioinformatic analysis

Fecal microbial DNA was extracted with the MagNA Pure LC DNA isolation kit III (Bacteria, Fungi) from (Roche, Mannheim, Germany), according to the manufacturer’s instructions. 16S rRNA gene amplicons were obtained following the 16S Metagenomic Sequencing Library Preparation Illumina protocol (15044223). The forward primer (5′-TCGTCGGCAGCGTCAGATGTGTATAAGAGACAGCCTACGGGNGGCWGCAG-3′) and the reverse primer (5′-GTCTCGTGGGCTCGGAGATGTGTATAAGAGACAGGACTACHVGGGTATCTAATCC-3′) were used to amplify by polymerase chain reaction (PCR) the V3-V4 hypervariable region of the 16S rRNA gene (Klindworth et al., 2013). After 16S rRNA gene amplification, the multiplexing step was performed using the Illumina Nextera XT DNA Library Preparation Kit (FC-131-1096, Illumina, Inc., United States). One μL of the PCR product was run on a Bioanalyzer DNA 1000 chips (Agilent, Santa Clara, CA, United States) to verify the size. The amplicon size on a Bioanalyzer trace is ~550 bp. After size verification, the libraries were sequenced using a 2 × 300 pb paired-end run [MiSeq Reagent kit v3 (MS-102-3001)] on a MiSeq Sequencer (Illumina, Inc., United States) according to the manufacturer’s instructions. Quality assessment of reads was performed using the prinseq-lite program (Schmieder and Edwards, 2011). The sequence data was analyzed using the QIIME2 pipeline (Caporaso et al., 2010). Denoising, paired-ends joining, and chimera depletion were performed starting from paired ends data using the DADA2 pipeline (Callahan et al., 2016). Taxonomic affiliations were assigned using the Naive Bayesian classifier integrated into QIIME2 plugins. The alpha diversity indexes (Shannon, Simpson, OBS, Chao1, and ACE) were calculated with the vegan R package (Oksanen et al., 2022) to find possible changes in fecal microbiota diversity in response to the dietary inclusion of olive cakes. Beta diversity was measured using the Bray-Curtis distance and plotted with the principal coordinates analysis (PCoA) to show further discrimination among the treatment groups.

### 2.4 Growth performance, nutrient digestibility, and slurry measurements

All animals used in this study were weighed at the beginning and the end of the experimental period, and the average daily gain (ADG) was calculated. Average daily feed intake (ADFI) was calculated by the difference between feed allowance and feed refusals over the experimental period. The feed conversion ratio (FCR) was calculated as the feed intake per kg BW gain, as reported by Ferrer et al. (2020).

At the end of the nutrient balance period, the feces collected from the 24 animals housed in metabolism pens were pooled per animal and kept at −20°C until analyses of energy and N. The feces and urine produced during the emissions period were pooled per animal to elaborate artificial slurry. Once reconstituted, the individual artificial slurries were analyzed for pH, SCFA concentration and potential gas emissions. Potential ammonia (NH_3_) emissions from slurries were measured in two replicates per animal following the procedure described by Beccaccia et al. (2015). Briefly, two plastic bottles per animal were filled with 0.5 kg of slurry and connected to two absorption flasks (impingers) containing 100 mL of sulfuric acid 0.1 N and an air pump that allowed to drive the air going through a line at a constant airflow rate of 1.2 L/min. Sulfuric acid captured the NH_3_ in the air extracted from each plastic bottle. The amount of NH_3_ in the acid was determined according to the Standard Methods Committee of the American Public Health Association, (2023) 4500 NH_3_-D procedure using a detection electrode (Orion high-performance NH_3_ electrode, model 9512HPBNWP, Thermo Scientific, Waltham, MA, United States).

Potential methane (CH_4_) emissions from slurry were measured as the biochemical CH_4_ potential (BMP) described by Angelidaki et al. (2009) and Beccaccia et al. (2015). The inoculum was obtained from an anaerobic wastewater treatment plant digester (Sagunto, Spain). Each slurry was mixed with the inoculum at a ratio of 1:1 on an organic matter (OM) basis and allocated in 120 mL bottles (3 replicates per slurry). Each one contained 40 mL of inoculum and a variable amount of slurry. The BMP assay also included three blank samples containing 40 mL of inoculum. Before incubation, bottles were flushed with N_2_, sealed with butyl rubber stoppers and aluminum crimps, and incubated (35°C) for 102 days. The pressure of the gas contained in bottles and CH_4_ concentration was quantified two or three times a week with a manometer (Delta Ohm, HD 9220, Padova, Italy) equipped with a split injector and a focus gas chromatograph (Thermo, Milan, Italy) with a flame ionization detector. Biochemical CH_4_ potential (B_0_) was expressed as the accumulative CH_4_ production per gram of OM.

### 2.5 Chemical analyses

OC and feeds were analyzed for dry matter (DM), ash, crude fat, N, energy, and total dietary fiber (TDF) content according to the Association of Official Analytical Chemists (AOAC, 2019) procedures. Total N was measured by combustion, using Leco equipment (model FP-528, Leco corporation, St. Joseph, MI, United States), and protein content was estimated as N content × 6.25 (AOAC 2000, method 986.06). The energy was determined using an isoperibolic bomb calorimeter (Parr 6400, Parr Instruments Co., Moline, IL, United States). Neutral detergent fiber (NDF), acid detergent fiber (ADF) and acid detergent lignin (ADL) in OC (PDOC and COC) were analyzed sequentially following Van Soest et al. (1991) procedure using the filter bag system (Ankom technology corp. Macedon, NY, United States) and expressed without residual ash. In addition, the contents in insoluble and soluble fiber were estimated as the NDF corrected by the protein content in the residue (insoluble) and the difference between TDF and NDF corrected by the protein content in the residue (soluble), respectively, as described in Beccaccia et al. (2015).

Main metabolite concentrations in samples of OC and experimental feeds were extracted following a previously described procedure by Belloumi et al. (2023). Briefly, phenolic compounds were analyzed on an Ultra-Performance Liquid Chromatography (UPLC) system coupled to a Fortis triple quadrupole (TSQ) mass spectrometer (Thermo Fisher Scientific, Madrid, Spain), in an Acquity Premier HSS T3 C18 (100 × 2.1 mm, 1.8 μm, Waters) column. The mobile phase was acetonitrile (A) and 0.1% formic acid (B) in a linear gradient of 10 min at 0.3 mL/min, starting with 10% A and reaching 100% A in 4 min, and then back to the initial conditions. Molecule detection was carried out using a diode array detector and spectrometry. Chromatograms were recorded at 200–400 nm absorbance, and mass analysis was run under electrospray positive and negative modes in a full scan from 100 to 850 m/z. Chromeleon, 7.3 chromatography data system software, was used for data treatment. Compounds were identified by comparing them to an authentic standard (hydroxytyrosol, oleacein, p-coumaric acid, tyrosol and verbascoside) and quantified using an external calibration curve with them. Three replicates per sample were analyzed. Hydroxytyrosol (TargetMol), oleacein (PhytoLab), tyrosol (TargetMol), and verbascoside (Life Science) were obtained from Cymit Química S.L. (Barcelona, Spain), and p-coumaric acid was purchased from Sigma Aldrich (Merck KGaA, Darmstadt, Germany). Four concentrations were prepared to carry out each external calibration curve: 0.05; 0.02; 0.01; and 0.005 mg/mL. Stock solutions of each compound at a concentration of 0.5 mg/mL were prepared using dimethyl sulfoxide, except for p-coumaric acid, which was dissolved in methanol. All standards and samples were filled into High performance liquid chromatography (HPLC) brown glass vials and sealed correctly to protect the solutions from light and evaporation.

Feces from the balance period were dried and ground to 1 mm pore size and analyzed for DM, energy and N content following the same methodology as for the diets. Slurry pH was measured using a glass electrode (Crison Basic 20+, Crison, Barcelona, Spain). The SCFA profile in the slurry was analyzed with gas chromatography equipped with a flame ionization detector (HP 68050 series Hewlett Packard, United States), as Jouany (1982) described, with the addition of an internal standard (4-metil valeric).

### 2.6 Calculations and statistical analysis

For the microbiota statistical analysis, the pipeline core was run using the programming language R (R Development Core Team, 2012). The Kruskal-Wallis test was used to determine significant differences in alpha-diversity indexes among the groups. PCoA plot was carried out using the FactoMineR R package (Lê et al., 2008). The beta diversity was evaluated by permutational multivariate analysis of variance (PERMANOVA), and significance was determined through 999 permutations. The differential abundance analysis among the groups was performed using the ALDEx R package (Fernandes et al., 2013).

The coefficient of total tract apparent digestibility (CTTAD) of DM, energy and N in experimental diets were calculated using the following equation:


$$\mathrm{CTTAD}\;\left(\%\right)=\left[\frac{N_{\mathrm{input}}-N_{\mathrm{output}}}{N_{\mathrm{input}}}\right]*100$$

Where: *N*_input_ is the amount of the nutrient (DM, energy, and *N*) ingested, and *N*_output_ is the quantity of the nutrient (DM, energy and N) excreted in feces (Kong and Adeola, 2014).

Differences in growth performance, CTTAD, SCFA concentrations, and gas emission among treatment groups were analyzed using the GLM procedure of SAS^®^ (Statistical Analysis System) System Software (Version 9.1, SAS Institute Inc., Cary, North Carolina, EEUU), with the dietary treatment as the main class effects and batch as a blocking factor.

Additionally, a volatility test analysis was performed using a QIIME2 plugin (q2-longitudinal) to assess the relationship among animal growth performance, nutrient balance and gas emission data, and changes in bacterial communities at the genus level. The genera most closely associated with each trait were chosen based on their *p*-value (*p* < 0.05) and r-coefficient.

## 3 Results

The analyzed chemical composition of PDOC and COC revealed slight differences in their nutritional content (Table 1). COC exhibited a higher ash and crude fat content than PDOC. On the other hand, PDOC showed substantially higher levels of total dietary fiber, NDF, ADF and insoluble fiber compared to COC. However, COC showed a greater soluble fiber content than PDOC. Regarding phenolic compounds, both sources of OC (PDOC and COC) contained the same compounds, which were hydroxytyrosol, tyrosol, verbascoside, oleacein and p-coumaric acid, with COC showing slightly higher levels of all of them compared to PDOC. Table 2 shows the ingredients and chemical composition of the experimental diets. Diets with OCs (20PDOC and 20COC) had higher crude fat, gross energy and fiber levels than the C diet. However, the crude protein and metabolizable energy content of diets with OCs was slightly lower than that of C diet. Regarding phenolic compounds, the 20PDOC and 20COC diets contained hydroxytyrosol, tyrosol, verbascoside, oleacein, and p-coumaric acid, whereas no one of these compounds was detected in the C diet.

### 3.1 Fecal bacterial composition

The V3-V4 hypervariable region of the 16S rRNA gene was sequenced from 60 fecal samples of crossbred pigs collected at two time points (30 before and 30 after providing the experimental diets). The average of high-quality sequences per sample was 32,284. Detailed information for each analyzed sequence can be found in (Supplementary Table S1).

**Figure 1:**
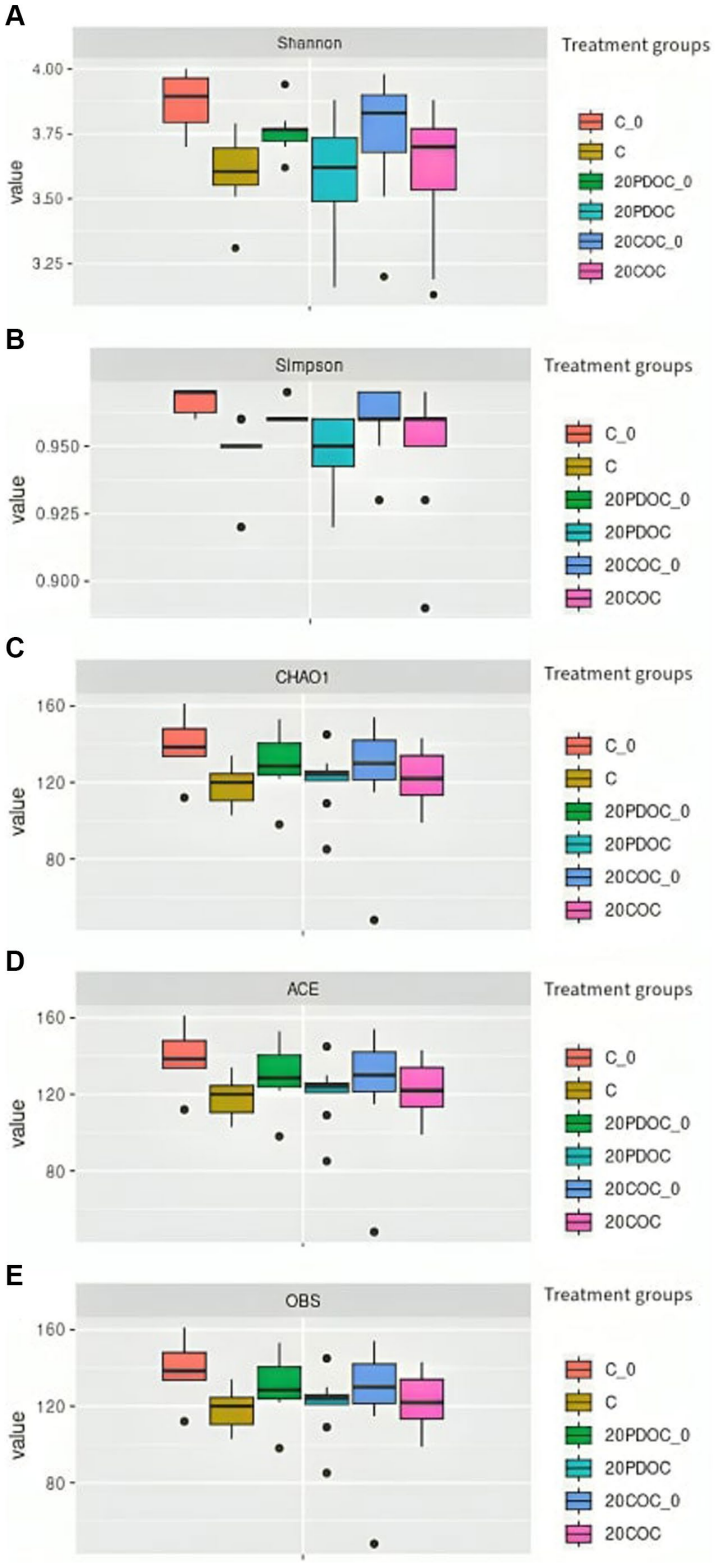
Alpha diversity indexes boxplots across dietary treatment groups at the beginning and at the end of the experiment. **(A)** Shannon, **(B)** Simpson, **(C)** CHAO1, **(D)** ACE, **(E)** OBS. Treatments groups are: C_0 = control group at the beginning of the experiment, C = control group at the end of the experiment, 20PDOC_0 = 20PDOC group at the beginning of the experiment, 20PDOC = 20PDOC group at the end of the experiment, 20COC_0 = 20COC group at the beginning of the experiment and 20COC = 20COC group at the end of the experiment.

**Figure 2:**
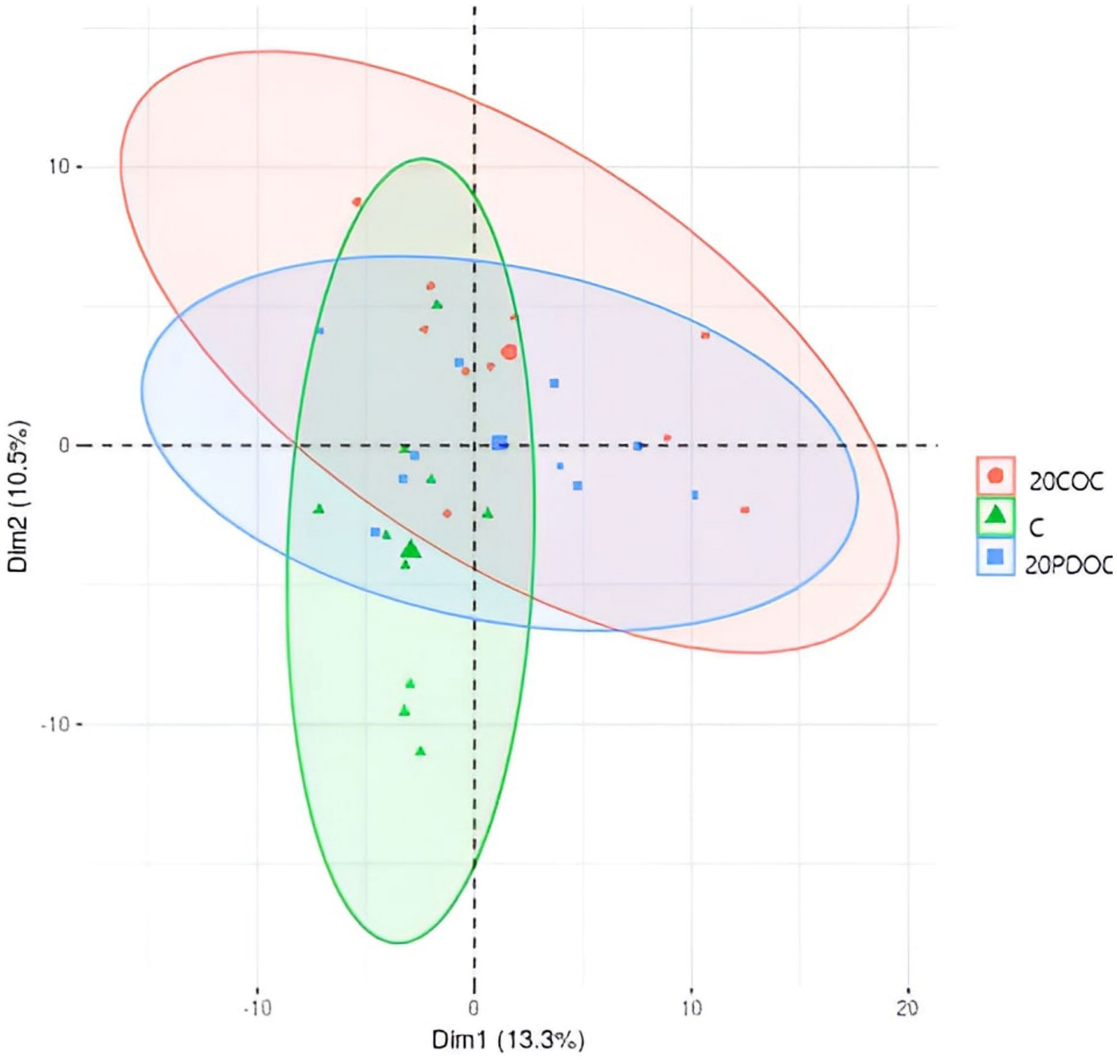
Principal coordinate analysis at the end of the experiment. Treatment groups are: C = control group; 20PDOC = group fed an experimental diet with 20% of partially defatted olive cake; 20COC = group fed an experimental diet with 20% of cyclone olive cake.

Figure 1 shows the alpha diversity indexes (richness and evenness) calculated in this study. No significant differences were found among the three groups of treatment in richness (OBS, Chao1, and ACE) and evenness indexes (Shannon and Simpson), neither at the onset nor at the end of the experiment. A non-significant decrease in diversity was observed in all treatment groups at the end of the experimental period. Regarding the beta diversity, the PCoA plot showed clear separation among treatments (Figure 2), showing animals from group C a significant discrepancy from animals from the 20PDOC group (PERMANOVA, *p* = 0.021) and animals from the 20COC group (PERMANOVA, *p* = 0.002).

**Figure 3:**
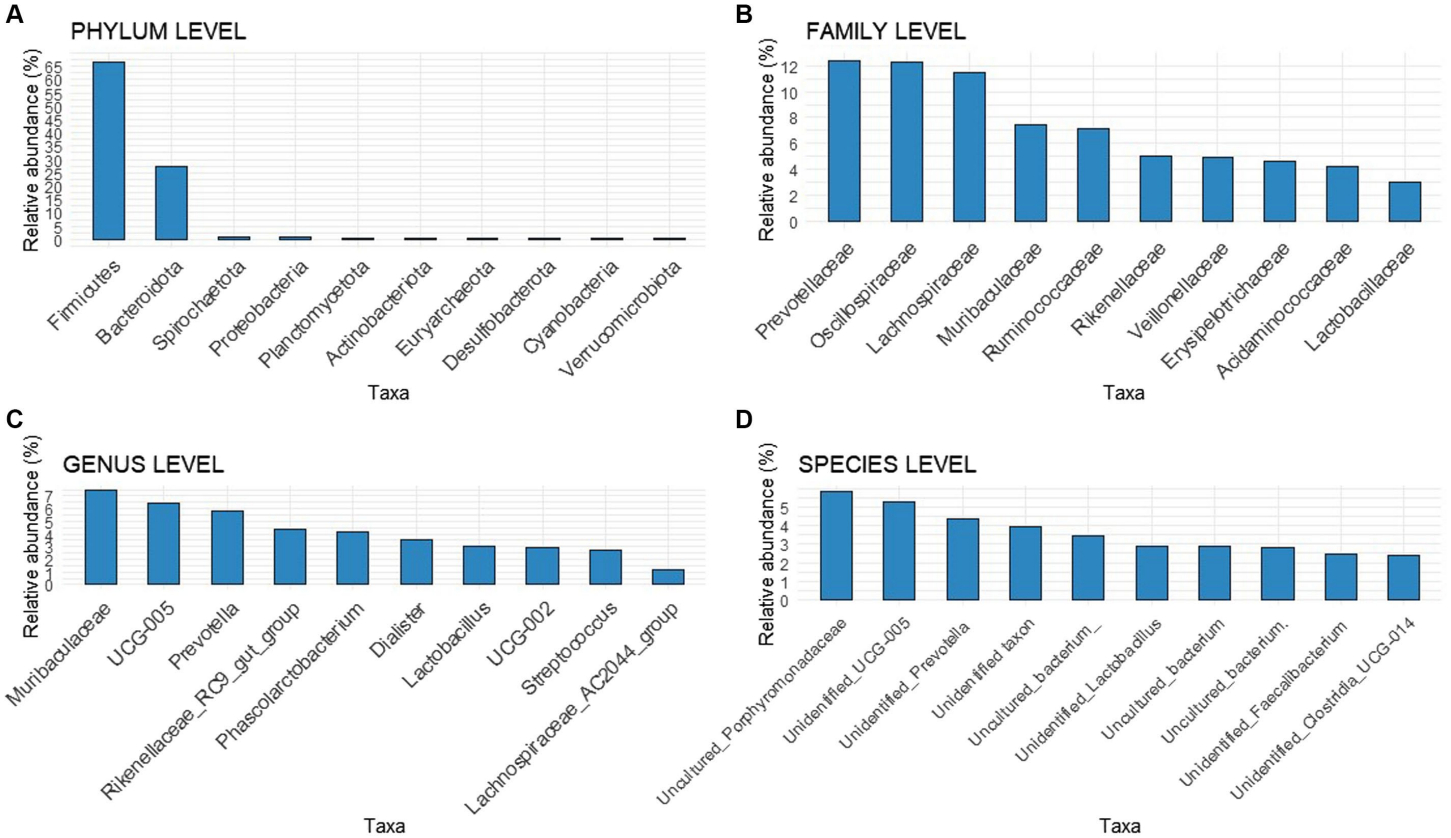
Bar chart with relative abundances of the Top 10 taxa at the onset of the experiment at different levels **(A)** Phylum level, **(B)** Family level, **(C)** Genus level, **(D)** Species level.

The relative abundances of taxa in the fecal microbiota were analyzed before and after the experimental feeds and provided. At the beginning of the experiment, no differences were observed among animals at the phylum, family, genus, or species levels (Figure 3). Firmicutes and Bacteroidota were the two most prominent taxa at the phylum level, accounting for 66.70 and 27.57% of the total, respectively. At the family level, Prevotellaceae was the most abundant bacteria, with a relative abundance of 12.4%. At the genus level, the main genera found were an unknown genus from the Muribaculaceae (7.41%) family, a genus-level group *Oscillospiraceae_UCG-005* (6.38%), and *Prevotella* (5.78%), followed by *Rikenellaceae_RC9_gut_group* (4.33%) and *Phascolarctobacterium* (4.13%). At the species level, an unknown species-level group *uncultured_Porphyromonadaceae* (family Muribaculaceae) was the most dominant taxon (5.87%), followed by an unidentified taxon from the genus-level group *Oscillospiraceae_UCG-005* genus (5.30%), and an unidentified taxon from the *Prevotella* genus (4.41%).

**Figure 4:**
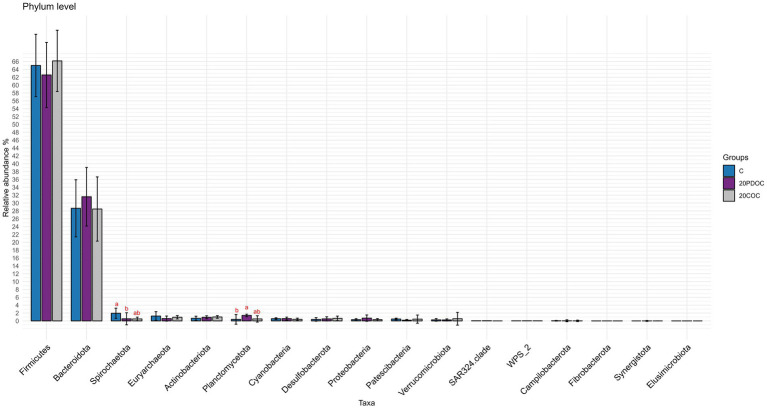
Bar chart with abundances of all taxa at the end of the experiment at phylum level. C = control group; 20PDOC = group fed an experimental diet with 20% of partially defatted olive cake; 20COC = group fed an experimental diet with 20% of cyclone olive cake. Standard deviation is provided as error bars. Means within a taxa without a common letter differ (*p* < 0.05).

**Figure 5:**
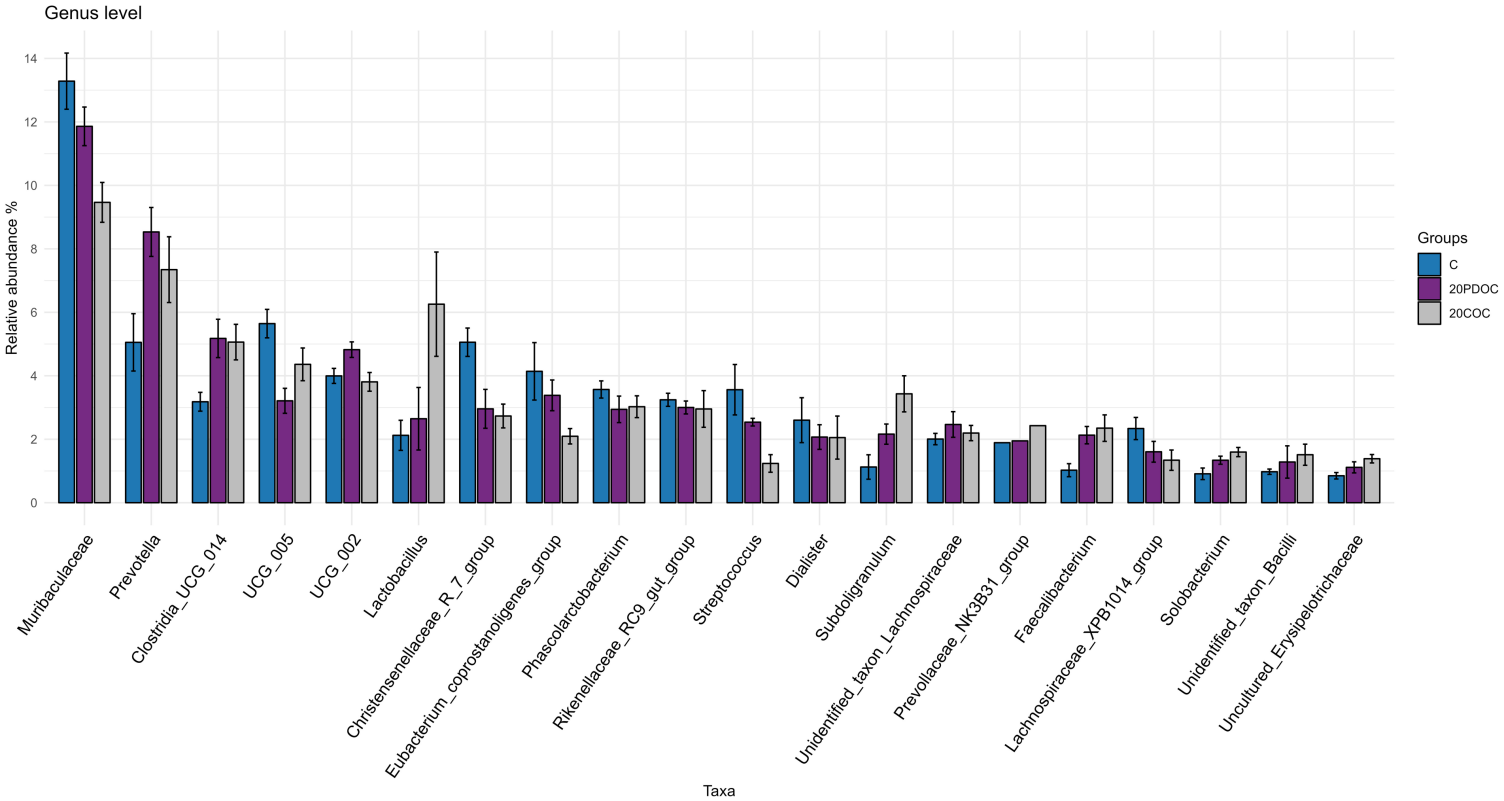
Bar chart with abundances of the Top 20 taxa at the end of the experiment at genus level. C = control group; 20PDOC = group fed an experimental diet with 20% of partially defatted olive cake; 20COC = group fed an experimental diet with 20% of cyclone olive cake. Standard deviation is provided as error bars.

**Figure 6:**
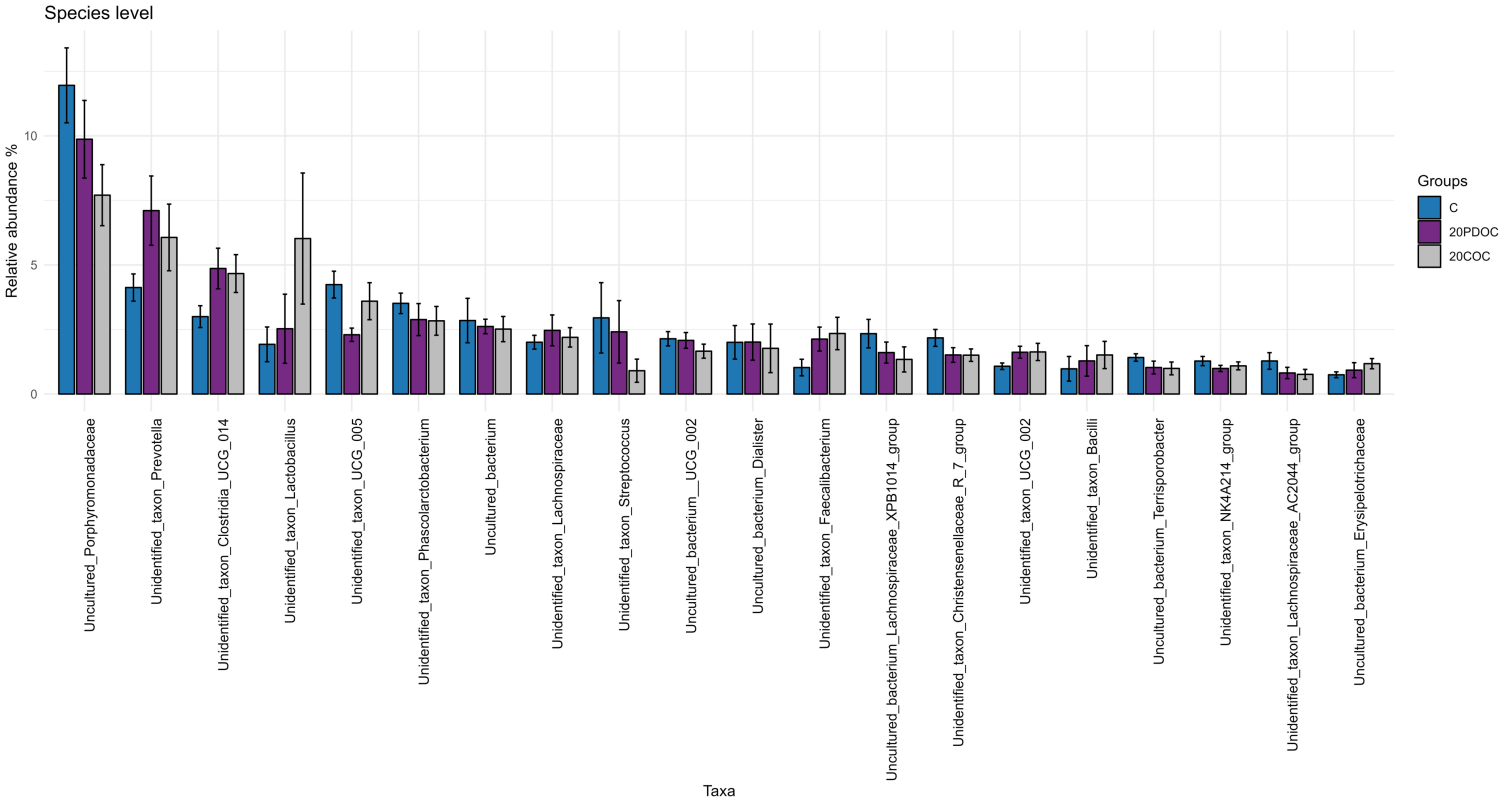
Bar chart with abundances of the Top 20 taxa at the end of the experiment at species level. C = control group; 20PDOC = group fed an experimental diet with 20% of partially defatted olive cake; 20COC = group fed an experimental diet with 20% of cyclone olive cake. Standard deviation is provided as error bars.

**Figure 7:**
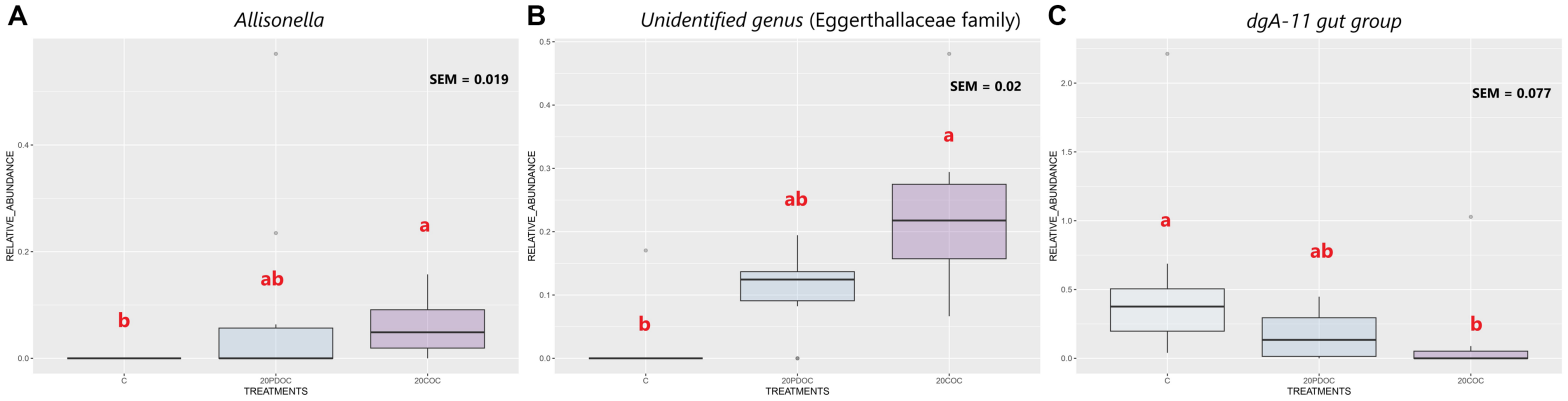
Boxplots of the discriminant genera among the treatment groups at the end of the experiment. Treatment groups are: C = control group; 20PDOC = group fed an experimental diet with 20% of partially defatted olive cake; 20COC = group fed an experimental diet with 20% of cyclone olive cake. SEM = standard error of the mean. Means within a taxa without a common letter differ (*p* < 0.05).

**Figure 8:**
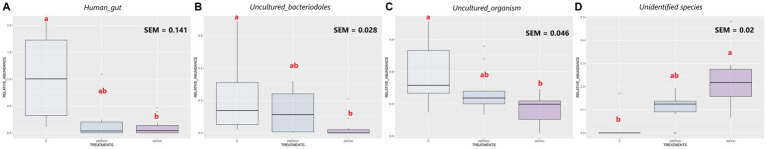
Boxplots of the most discriminant species among the treatment groups at the end of the experiment. Treatment groups are: C = control group; 20PDOC = group fed an experimental diet with 20% of partially defatted olive cake; 20COC = group fed an experimental diet with 20% of cyclone olive cake. SEM = standard error of the mean. Means within a taxa without a common letter differ (*p* < 0.05).

At the end of the experiment, the top 20 taxa with the highest relative abundances at different taxonomic levels are presented: phylum level (Figure 4), genus level (Figure 5), and species level (Figure 6). At the phylum level, Firmicutes and Bacteroidota remained the predominant taxa across the three groups, comprising more than 93% of the total abundance. However, the differential abundance analysis performed using ALDEx revealed that Spirochaetota was significantly (*p* < 0.05) more abundant in the C group than in the 20PDOC group, contrary to Planctomycetota, which was significantly more abundant (*p* < 0.05) in the 20PDOC group compared to the C group, with the 20COC group showing intermediate levels for both. At the genus level, the dominant genera in the C group were similar to those found before providing the experimental diets, with an unknown genus from the Muribaculaceae family (13.28%) as the dominant one, followed by the genus-level group *Oscillospiraceae_UCG-005* (5.64%) and *Prevotella* (5.05%). In animals fed with OC, the dominant genus was also an unknown genus from Muribaculaceae family (11.86% in 20PDOC and 9.46% in 20COC). However, ALDEx analysis revealed significant differences in bacterial relative abundance between OC and C animals at the genus level at the end of the experiment (Figure 7). In this regard, *Allisonella* and an unidentified genus from the Eggerthallaceae family were significantly (*p* < 0.05) enriched in the 20COC group compared to the C group. The group 20PDOC showed intermediate abundances of these genera. On the contrary, the genus-level group *dgA-11_gut_group* (family Rikenallaceae) was more (*p* < 0.05) abundant in the C group than in the 20COC group, with the animals fed PDOC showing intermediate values. On the other hand, although not significant, pigs fed with OC (20PDOC and 20COC) showed a slightly higher representation of the *Prevotella* genus (8.53% in 20PDOC and 7.34% in 20COC) and *Clostridia-UCG-014* (5.18% in 20PDOC and 5.06% in 20COC) compared to pigs fed with the C diet (*Prevotella* = 5.05% and *Clostridia-UCG-014* = 3.18%). In addition, pigs fed with the 20COC diet showed a slight increase in the abundance of *Lactobacillus* compared to pigs from C and 20PDOC groups (6.25% in 20COC vs. 2.12% in C and 2.64% in PDOC). At the species level, the most abundant taxon in all the groups was an unknown species-level group *uncultured_Porphyromonadaceae* from the Muribaculaceae family (11.95% in C, 9.86% in 20PDOC, and 7.70% in 20COC). An unknown bacterium belonging to the *Prevotella* genus was the second most abundant in the 20PDOC and 20COC groups (7.10% in 20PDOC and 6.06% in 20COC). The discriminant taxa among the three groups at the species level are shown in Figure 8. *Human_gut* (a species-level group belonging to the family Christensenellales)*, Uncultured_organism* (a species-level group belonging to the family Anaerovoracaceae), and *Uncultured Bacteroidales* (a species-level group belonging to the family Rikenallaceae) were more (*p* < 0.05) abundant in the C group than in the 20COC group, while the 20PDOC group showed intermediate levels. Conversely, an unidentified taxon from the Eggerthellaceae family was significantly (*p* < 0.05) enriched in the 20COC group compared to the C group (Figure 8).

### 3.2 Growth performance, nutrient balance, and slurry measurements

The effects of dietary treatments on growth performance, nutrient digestibility, and gas emission are presented in Table 3. Pigs from the C group showed greater (*p* < 0.05) final BW and ADG compared to pigs from 20PDOC and 20COC groups. However, there was no difference in ADFI among treatment groups. As a result, FCR was significantly lower (*p* < 0.05) in the C group than in the 20PDOC and 20COC2 groups. The digestibility of energy and N was higher (*p* < 0.05) in the C group compared with groups 20PDOC and 20COC. Regarding slurry gas emission, potential NH_3_ emission was higher (*p* < 0.05) in the C group compared to 20PDOC and 20COC groups, and higher (*p* < 0.05) in 20PDOC animals than in 20COC animals. Furthermore, potential CH_4_ emission was significantly higher (*p* < 0.001) in the C group compared to the groups of animals fed with OC (20PDOC and 20COC).

**Table 3:** Effect of the dietary treatments on growth performance, nutrient digestibility, and gas emission.

	Treatments			SEM	*p*-value
	C	20PDOC	20COC		
**Growth performance**					
Initial body weight, kg^1^	54.2	53.8	54.2	0.962	0.949
Final body weight, kg^1^	75.2^a^	69.9^b^	69.7^b^	1.68	0.049
Average daily gain, kg/d^1^	0.99^a^	0.76^b^	0.73^b^	0.05	0.004
Average daily feed intake, kg/d^2^	2.07	2.04	1.91	0.11	0.541
Feed conversion ratio, g/g^1^	2.12^b^	2.72^a^	2.72^a^	0.17	0.033
**Coefficient of digestibility, %** ^2^					
Energy	90.4^a^	78.0^b^	78.9^b^	0.63	<0.001
Crude protein	87.9^a^	78.3^b^	75.4^b^	0.96	<0.001
**Gas emission** ^2^					
Ammonia, g/kg slurry	2.27^a^	1.38^b^	1.06^c^	0.083	<0.001
Methane, mL/g organic matter	301^a^	201^b^	236^b^	13.0	<0.001

C = control diet; 20PDOC = experimental diet with 20% of partially defatted olive cake in replacement of the energetic part of the control diet; 20COC = experimental diet with 20% of cyclone olive cake in replacement of the energetic part of the control diet; SEM: standard error of means; ^a,b,c^ Means in the same row with different superscripts differ statistically (*p* < 0.05).

1 *n* = 10.

2 *n* = 8.

In the case of SCFA, Table 4 shows the results of the SCFA profile per treatment. Pigs from the 20COC group showed higher (*p* < 0.05) levels of total SCFA, acetic acid, and butyric acid compared to the C group. The pigs from the 20PDOC group had intermediate levels. Both groups with OC (20PDOC and 20COC) presented higher (*p* < 0.05) levels of caproic acid than the C group, and the 20PDOC group had higher (*p* < 0.05) levels of heptanoic acid than the C group, with intermediate values shown for 20COC. No significant differences were observed for propionic, isobutyric, isovaleric, and valeric acids among the animals from the three groups.

**Table 4:** Effect of the dietary treatments on slurry volatile fatty acid concentration (mg/g slurry).

Short-chain fatty acids^1^	Treatments			SEM	*p*-value
	C	20PDOC	20COC		
Total SCFA	84.3^b^	104^ab^	112^a^	6.55	0.024
Acetic acid	64.5^b^	81.6^ab^	85.1^a^	5.51	0.037
Propionic acid	9.69	9.98	11.2	1.11	0.582
Isobutyric acid	0.911	1.05	1.01	0.095	0.571
Butyric acid	5.67^b^	7.52^ab^	9.84^a^	1.08	0.044
Isovaleric acid	1.78	1.67	1.84	0.117	0.584
Valeric acid	1.47	1.45	1.36	0.167	0.867
Caproic acid	0.262^b^	0.616^a^	0.722^a^	0.091	0.006
Heptanoic acid	0.063^b^	0.147^a^	0.130^ab^	0.022	0.035

C = control diet; 20PDOC = experimental diet with 20% of partially defatted olive cake in replacement of the energetic part of the control diet; 20COC = experimental diet with 20% of cyclone olive cake in replacement of the energetic part of the control diet; SEM: standard error of means; ^a,b,c^ Means in the same row with different superscripts differ statistically (*p* < 0.05); SCFA, short-chain fatty acids.

1 *n* = 8.

### 3.3 Volatility analysis

Table 5 displays the volatility analysis results, with the correlation coefficients (*r*-value) and corresponding significance (*p*-values) of the relationships between the performance, digestibility and slurry variables studied and bacterial genera found to be significant or near to significance. The variables were categorized into four groups: growth performance (ADG, ADFI, and FCR), nutrient balance (digestibility), slurry characteristics (pH, SCFA) and potential gas emission (NH_3_ and CH_4_ emission). Growth performance variables were not significantly correlated with specific bacterial genera, so they were not represented in Table 5. In terms of digestibility, the CTTAD of energy was positively correlated (*p* < 0.05) with *Uncultured_Bacteroidales* and *Unculured_Selenomonadaceae*, while the CTTAD of DM was positively correlated (*p* < 0.05) with the genus-level groups *Uncultured_Bacteroidales* and *Clostridium_sensu_stricto_6*. Under the emission category, the total SCFA in the slurry showed a significant (*p* < 0.05) positive correlation with the genera *Monoglobus* and *Desulfovibrio*, and the acetic acid had a positive (*p* < 0.05) correlation with the genera *Monoglobus* and *Treponema*. Also, trends (*p* = 0.062) were detected for a positive correlation between heptanoic acid and the genus-level groups *Clostridia_UCG-014* and *Clostridia_UCG-007*, and between N-valeric acid and *[Eubacterium]_eligens_group* and *Prevotellaceae_NK3B31_group*. In contrast, a negative correlation trend (*p* = 0.055) was observed between the pH value and the genera *Solobacterium* and *Oscillospira*.

**Table 5:** Volatility analysis results.

Parameters	*r*-value	*p*-value	Important genera
**Nutrient balance**			
Coefficient of digestibility of gross energy	0.947	0.014	*Unculured_Bacteroidales*; *Unculured_Selenomonadaceae*
Coefficient of digestibility of dry matter	0.924	0.024	*Unculured_Bacteroidales*; *Clostridium_sensu_stricto_6*
**Slurry characteristics**			
Total SCFA	0.921	0.026	*Monoglobus*; *Desulfovibrio*
Acetic acid	0.985	0.002	*Monoglobus*; *Treponema*
Heptanoic acid	0.858	0.062	*Clostridia_UCG-014*; *UCG-007*
N-valeric acid	0.847	0.069	*[Eubacterium]_eligens_group*; *Prevotellaceae_NK3B31_group*
pH	−0.87	0.055	*Solobacterium*; *Oscillospira*

SCFA, short-chain fatty acids.

## 4 Discussion

Recently, there has been a growing interest in exploring the impact of incorporating fibrous by-products into pig diets due to their positive impact on the sustainability of the livestock sector and their potential effects on gut bacterial composition and health. In the present study, the effect of including OC, a by-product of the olive oil industry, in pig diets on fecal microbiota composition was investigated. Also, growth performance, nutrient digestibility, and gas emission from pigs fed with OC were determined. Although these parameters were not the objective of this study, they were included with the aim of studying their potential correlation with selected microbial taxa, since these aspects are relevant to pig production.

In the design of the diets for the present study, the two OCs were included in the respective diets to replace the energetic part of the diet, maintaining the mineral fraction of the diet constant. This design means that OCs are the only source of variability among diets and allows the study of the effects of OCs on gut microbiota composition, avoiding confounding effects due to changes in the proportion of other ingredients in the diet and deficiencies in micronutrients. However, this design causes differences in macronutrient composition among diets (greater fat, fiber and gross energy and lower protein in OC diets) that led to differences in energy and protein digestibility, which are the main dietary fractions affecting growth performance. In this regard, animals fed with OC showed lower energy and protein digestibility coefficients and, therefore, an impaired growth performance (lower ADG and higher FCR) compared with C animals. Thus, these results were expected in the context of the present study. It is well known that fiber, and especially insoluble fiber, can reduce nutrient digestibility in pigs (Acosta et al., 2020). OCs are characterized by a high insoluble fiber content in their composition, which is one of the main factors affecting nutrient digestibility in the present study. Regarding gas emission, differences observed among treatments are probably due to changes in the physicochemical properties of the slurries generated caused by OCs. Slurries from animals fed OC in the present study had higher DM concentration and a lower pH than those of C animals, and these parameters can affect NH_3_ and CH_4_ emission per unit of slurry (Piquer et al., 2022).

In terms of gut microbiota, the impact of dietary energy has scarcely been addressed and the impact of protein is not clear in the literature (Zhou et al., 2016; Pollock et al., 2019), probably because the same dietary energy and protein levels can be achieved by several combinations of macronutrients. However, several studies suggest that the concentration of specific macronutrients, such as fiber or fat, and other dietary components, such as phenolic compounds or oleic acid in the diet can have a highly marked effect on fecal bacterial composition (Pu et al., 2016; Jha et al., 2019; Moorthy et al., 2020; Yang et al., 2020). Dietary fiber escapes digestion by host endogenous enzymes in the proximal small intestine and is known to serve as a substrate for bacteria in the large intestine. An extensive number of studies confirm that its presence and source can influence the diversity and abundance of microbial species and promote gut health (Jha et al., 2019). Compared to other nutrients, the digestibility of fat is usually high. However, it has been described that indigestible fats might act as a nutritional source for bacteria, altering gut microbiota composition, with the most significant changes observed in the small intestine (Yang et al., 2020). Also, other dietary components characteristic in olive by-products, such as polyphenols and monounsaturated fatty acids (MUFA) as oleic acid, have been related to changes in gut microbiota composition. Polyphenols are naturally occurring phenolic compounds found in plant-based foods, have prebiotic properties, and can selectively promote the growth and activity of beneficial gut bacteria (Moorthy et al., 2020). On the other hand, the consumption of MUFAs-rich diets have showed positive health effects related to increases in the gut microbiota diversity of healthy and unhealthy humans at risk of metabolic syndrome (Pu et al., 2016).

In the present study, the changes in these components among diets are caused by OCs, since these are ingredients rich in fat, fiber (particularly insoluble fiber), phenolic compounds, and oleic acid. Thus, we can assume that the observed changes in the fecal microbiota composition are mainly due to OCs as a whole since the singular effects of the different nutrients cannot be separated.

In the present study, including olive by-products in pig diets did not result in differences in the richness and biodiversity of the fecal microbiota. However, results on beta diversity, which quantifies the dissimilarity among microbial communities, showed a clustering trend among the different groups, indicating that the inclusion of OC in the diet influenced the specific composition of the fecal microbiota. These results are consistent with previous studies conducted in humans (Rocchetti et al., 2022), cows (Russo et al., 2023), sows (Sánchez et al., 2022), and broilers (Dedousi et al., 2023), highlighting olive by-products’ ability to modulate the gut microbiota composition.

The phyla Firmicutes and Bacteroidetes were predominant in all pigs, before and after the nutritional challenge. These results are consistent with previous research conducted in pigs by Ramayo-Caldas et al. (2016) and Yang and Zhao (2021). Indeed, the presence of Firmicutes and Bacteroidetes in the gastrointestinal tracts of pigs plays a vital role in promoting overall animal health (Kim et al., 2011). After 21 days of feeding the experimental diets, the 20PDOC group showed a significant decrease in the relative abundance of Spirochaetota and an increase in the relative abundance of Planctomycetota at the phylum level compared with the C group. Spirochaetota, a phylum consisting of a wide array of pathogenic microorganisms, is frequently found in the intestines of pigs (Millar et al., 2011). With its diverse nature encompassing over 90 identified species, this phylum is characterized by containing highly prevalent disease-causing agents (Gupta et al., 2013). On the other hand, Planctomycetota has been described as a promoter of the good functioning of the immune system (Fuerst and Sagulenko, 2013). Therefore, the dietary inclusion of PDOC has the potential to positively impact fecal bacterial composition, highlighting the potential benefits of PDOC supplementation in promoting a healthier gut environment in pigs. At the genus level, the results of our study revealed a significant enrichment of an unidentified genus from the Eggerthellaceae family and the genus *Allisonella* in the 20COC group when compared to the C group, whereas the 20PDOC group showed intermediate levels. Similarly, Rocchetti et al. (2022) found that including phenolic olive leaf extract in feeds increased the proportion of Eggerthellaceae in humans. Eggerthellaceae is a polyphenol-degrading family with a positive role in the gut microbiota due to their capacity to release bioactive molecules from dietary polyphenols that can positively impact the microbiota, reducing pathogens and improving host health (Rodríguez-Daza et al., 2021). Thus, the higher content of phenolic compounds found in COC compared with PDOC and C diets could have induced these differences. On the other hand, the *Allisonella* genus has been strongly correlated with caproic acid production (Che et al., 2019). Caproic acid has antimicrobial potential and stimulates the immune system of humans and animals (Trachsel et al., 2022). On the contrary, fecal samples of C pigs showed a significantly (*p* < 0.05) greater abundance of the Bacteroidales *dgA-11_gut group* than those of 20COC pigs, with the PDOC group displaying intermediate levels. Previous research conducted by Deng et al. (2022) revealed a positive association between this group and the production of acetic acid, suggesting a possible enhancement in lipid metabolism, which does not agree with the greater concentration of acetic acid found in slurry from animals fed diets with OC (20PDOC and 20COC) in the current study. At the species level, *Human_gut*, *Uncultured_organism* (belonging to *Family_XIII_AD3011_group*), and *Uncultured Bacteroidales* (belonging to *dgA-11_gut_group*) were enriched in the C group compared with the 20COC group, while 20PDOC animals showed intermediate levels. According to Wang et al. (2022), *Family_XIII_AD3011_group* had a higher abundance in pigs susceptible to porcine reproductive and respiratory syndrome. As reported by Zhang et al. (2021), this bacterial group might induce inflammation and contribute to excessive insulin secretion, leading to the development of hypoglycemic symptoms. No information was found in the literature on the effects of the species-level group *Human_gut* on animals’ or humans’ health.

On the other hand, these changes in fecal microbiota composition could have provoked changes in the gut’s fermentative activity and fermentation products (such as SCFA). The results of the present study demonstrate that providing diets with OC to pigs can increase the production of SCFA, as Lindberg (2014) reported. SCFAs are mainly produced by bacterial fermentation of dietary fiber and can be an essential energy source in growing pigs. They can provide between 5 and 20% of the total available energy for pigs (Ashida et al., 2011). SCFAs are also involved in maintaining gut integrity (Yang and Zhao, 2021) and whole-body homeostasis. Our results also reveal that including OC in pig diets can increase the total concentrations of some individual SCFA (acetic and butyric acids) and medium-chain fatty acids (MCFA) such as caproic acid, and heptanoic acid. Therefore, despite the high lignification level (insoluble fiber) of the OC’s fiber fraction, its inclusion in pig diets can promote bacterial fermentation in the gut. Zhao et al. (2019) reported that the amount of SCFA produced by the gut microbiota was positively correlated with the insoluble dietary fiber content in fiber-rich ingredients, not with the soluble fiber. This can be explained by the fact that the concentration of SCFA is influenced not only by the chemical composition of the fiber but also by its physical characteristics and molecular structure (Zhao et al., 2020). In the present study, acetic, butyric, caproic and heptanoic acids were promoted by including OC in diets. Acetic acid is taken up mainly by the liver as a precursor for synthesizing fatty acids (Tan et al., 2021). On the other hand, numerous studies have detected a positive relationship between the fermentation of insoluble fiber sources and the butyrate concentration in the large intestine (Molist et al., 2010). It is known that butyric acid positively impacts gut health by improving gut morphology, the barrier function of the colonocytes (Bedford and Gong, 2018), and immunity (Kelly et al., 2015). Thus, butyric acid can be considered a biomarker of host health in pigs (Vasquez et al., 2022) and humans (Baldi et al., 2021). Caproic and heptanoic acids are MCFAs, and their relationship with fiber fermentation has been scarcely studied in the literature. Heptanoic acid has shown antimicrobial properties having a positive role in host health (Grilli et al., 2013). In the case of caproic acid, several studies demonstrate that, like other MCFAs, it exhibits potent antimicrobial and immunomodulatory activities (Trachsel et al., 2022). Therefore, OC in pig diets can potentially affect SCFA production, which can have potential effects on gut health and the immune system of the animals. However, these effects on SCFA are not the same in the two OCs studied. The effects on acetic and butyric acids were stronger in the case of COC, whereas the effects on heptanoic acid were greater in the case of PDOC. These differences could probably be attributed to differences in the chemical composition of PDOC and COC. In this regard, PDOC exhibited a greater lignin content compared to COC, and it is known that lignin is hardly fermented by bacteria in the intestines of pigs (Glitso et al., 1998).

With the aim of obtaining information on the relationship between pig performance variables, such as growth efficiency, nutrient digestibility and slurry gas emission, and the changes or fluctuations in the abundance of specific microbial groups, a volatility analysis was performed using all the animals. The abundance of the genus-level groups *Uncultured_Bacteroidales, Uncultured_Selenomonadaceae*, and *Clostridium_sensu_stricto_6* was positively associated with the CTTAD of energy and DM. This effect suggests that these groups likely contribute to the efficient degradation and utilization of dietary components, including fiber. According to Sun et al. (2015), *uncultured_Bacteroidales* were enriched in pigs fed a diet high in complex carbohydrates, suggesting an enriched biofunction of carbohydrate catabolism, whereas *Uncultured_Selenomonadaceae* are common inhabitants of the gut that use saccharides and lactate for growth (Dworkin et al., 2006). Additionally, some groups belonging to the *Clostridiaceae* family, such as *Clostridium_sensu_stricto_6*, have been positively linked to crude fiber digestibility and dietary fiber metabolism (Niu et al., 2015). On the other hand, the correlations observed between bacterial genera and the gas emission variables suggested potential associations between specific bacterial groups and the production of SCFAs and pH in the slurry, but not directly with gas (NH_3_ and CH_4_) emission. The abundance of the genera *Monoglobus*, *Desulfovibrio*, and *Treponema* were positively associated with total SCFA and acetic acid production. According to a report by Goris et al. (2021), the genus *Monoglobus* possesses the capacity for dietary fiber fermentation. This capability to digest complex polysaccharides will likely promote SCFA production, especially butyrate. Furthermore, the genera *Treponema* is involved in the degradation of cellulose, lignin, and resistant starch (Niu et al., 2015). These bacteria are also linked to the production of SCFAs (Li et al., 2019). *Desulfovibrio* is a sulfate-reducing bacteria related to sulfate metabolism and hydrogen sulfide production in the human gut. By interacting with other gut microbes, *Desulfovibrio* may indirectly impacts SCFA production (Carbonera et al., 2012). It is important to note that the specific associations between these bacterial genera and SCFA production in pigs need further investigation. The limited research available on these specific genera in the context of SCFA production and gas emission in pigs underscores the need for more targeted studies to fully understand their roles and mechanisms in the porcine gastrointestinal tract.

In conclusion, integrating agro-industrial by-products into livestock diets presents a valuable avenue for embracing the principles of the circular economy. By incorporating OC into pig diets at a rate of 20%, fecal microbiota composition can be shifted favorably, resulting in increased production of specific SCFAs in the slurry. As a result, fattening piglets may experience beneficial changes in their intestinal environment, which can lead to reduced inflammation and improved intestinal morphological development. Additionally, it seems that gut energy digestion can be associated with specific bacterial general, but this is not the case for gas emissions from slurry. More studies are needed to confirm this association with other environmental conditions and determine its implications in practice.

## Data availability statement

The datasets presented in this study can be found in online repositories (ReDivia; https://redivia.gva.es) and in the Supplementary material. For the 16S rRNA analysis, the raw sequences data are stored in European nucleotide archive (ENA); accession number PRJEB71070 https://www.ebi.ac.uk/ena/browser/view/PRJEB71070.

## Ethics statement

All experimental procedures were approved by the Universitat Politècnica de València Ethics Committee (with the registration number 2017/VSC/PEA/00166). A ll experiments were carried out following the recommendation in the ARRIVE guidelines (https://arriveguidelines.org).

## Author contributions

DB: Investigation, Methodology, Data curation, Formal analysis, Writing – original draft. PG-R: Conceptualization, Funding acquisition, Investigation, Methodology, Visualization, Writing – review & editing. SC: Conceptualization, Funding acquisition, Investigation, Methodology, Supervision, Visualization, Writing – review & editing. MPF: Conceptualization, Methodology, Resources, Validation, Writing – review & editing. MR-P: Data curation, Methodology, Software, Validation, Writing – review & editing. JG-G: Data curation, Software, Writing – review & editing. LP: Investigation, Methodology, Writing – review & editing. AIJ-B: Writing – review & editing. AB: Investigation, Methodology, Writing – review & editing. CC: Investigation, Writing – review & editing. AC: Conceptualization, Funding acquisition, Investigation, Methodology, Project administration, Resources, Supervision, Writing – review & editing.

## References

[ref1] Acosta J. A. Stein H. H. Patience J. F. (2020). Impact of increasing the levels of insoluble fiber and on the method of diet formulation measures of energy and nutrient digestibility in growing pigs. J. Anim. Sci. 98, 1–9. doi: 10.1093/jas/skaa130, PMID: 32315034 PMC7275632

[ref2] Angelidaki I. Alves M. Bolzonella D. Borzacconi L. Campos J. L. Guwy A. J.. (2009). Defining the biomethane potential (BMP) of solid organic wastes and energy crops: a proposed protocol for batch assays. Water Sci. Technol. 59, 927–934. doi: 10.2166/wst.2009.040, PMID: 19273891

[ref3] AOAC (2019). Official Methods of Analysis of the Association of Official Analytical Chemists: Official Methods of Analysis of AOAC International. 21st Edition, AOAC, Washington DC.

[ref4] Ashida H. Ogawa M. Kim M. Mimuro H. Sasakawa C. (2011). Bacteria and host interactions in the gut epithelial barrier. Nat. Chem. Biol. 8, 36–45. doi: 10.1038/nchembio.741 22173358

[ref5] Baldi S. Menicatti M. Nannini G. Niccolai E. Russo E. Ricci F.. (2021). Free fatty acids signature in human intestinal disorders: significant association between butyric acid and celiac disease. Nutrients 13:742. doi: 10.3390/nu13030742, PMID: 33652681 PMC7996737

[ref6] Beccaccia A. Calvet S. Cerisuelo A. Ferrer P. García-Rebollar P. De Blas C. (2015). Effects of nutrition on digestion efficiency and gaseous emissions from slurry in growing-finishing pigs. I. Influence of the inclusion of two levels of orange pulp and carob meal in isofibrous diets. Anim. Feed Sci. Technol. 208, 158–169. doi: 10.1016/j.anifeedsci.2015.07.008

[ref7] Bedford A. Gong J. (2018). Implications of butyrate and its derivatives for gut health and animal production. Anim. Nutr. 4, 151–159. doi: 10.1016/j.aninu.2017.08.010, PMID: 30140754 PMC6104520

[ref8] Belloumi D. Calvet S. Roca M. I. Ferrer P. Jiménez-Belenguer A. I. Cambra-López M.. (2023). Effect of providing citrus pulp-integrated diet on fecal microbiota and serum and fecal metabolome shifts in crossbred pigs. Sci. Rep. 13:17596. doi: 10.1038/s41598-023-44741-z, PMID: 37845279 PMC10579234

[ref9] Callahan B. J. McMurdie P. J. Rosen M. J. Han A. W. Johnson A. J. A. Holmes S. P. (2016). DADA2: high-resolution sample inference from Illumina amplicon data. Nat. Methods 13, 581–583. doi: 10.1038/nmeth.3869, PMID: 27214047 PMC4927377

[ref10] Caporaso J. G. Kuczynski J. Stombaugh J. Bittinger K. Bushman F. D. Costello E. K.. (2010). QIIME allows analysis of high-throughput community sequencing data. Nat. Methods 7, 335–336. doi: 10.1038/nmeth.f.303, PMID: 20383131 PMC3156573

[ref11] Carbonera F. Benefiel A. C. Alizadeh-Ghamsari A. H. Gaskins H. R. (2012). Microbial pathways in colonic sulfur metabolism and links with health and disease. Front. Physiol. 3:448. doi: 10.3389/fphys.2012.00448, PMID: 23226130 PMC3508456

[ref12] Chambers E. S. Viardot A. Psichas A. Morrison D. J. Murphy K. G. Zac-Varghese S. E. K.. (2015). Effects of targeted delivery of propionate to the human colon on appetite regulation, body weight maintenance and adiposity in overweight adults. Gut 64, 1744–1754. doi: 10.1136/gutjnl-2014-307913, PMID: 25500202 PMC4680171

[ref13] Che L. Hu Q. Wang R. Zhang D. Liu C. Zhang Y.. (2019). Inter-correlated gut microbiota and SCFAs changes upon antibiotics exposure links with rapid body-mass gain in weaned piglet model. J. Nutr. Biochem. 74:108246. doi: 10.1016/j.jnutbio.2019.108246, PMID: 31671360

[ref14] De Blas C. García-Rebollar P. Gorrachategui M. Mateos G. G. (2019). Tablas FEDNA de composición y valor nutritivo de alimentos para la fabricación de piensos compuestos. Madrid: Fundación Española para el Desarrollo de la Nutrición Animal.

[ref15] De Blas J. C. Gasa J. Mateos G. G. (2013). Necesidades nutricionales para ganado porcino: Normas FEDNA. Madrid: Fundación Española para el Desarrollo de la Nutrición Animal.

[ref16] De Blas J. C. Rodríguez C. A. Bacha F. Fernández R. Abad-Guaman R. (2015). Nutritive value of co-products derived from olive cake in rabbit feeding. World Rabbit Sci. 23, 255–282. doi: 10.4995/wrs.2015.4036

[ref17] Dedousi A. Kotzamanidis C. Kritsa M. Z. Tsoureki A. Andreadelli A. Patsios S. I.. (2023). Growth performance, gut health, welfare and qualitative behavior characteristics of broilers fed diets supplemented with dried common (*Olea europaea*) olive pulp. Sustain. For. 15:501. doi: 10.3390/su15010501

[ref18] Deng L. Chen S. Meng W. Zhou Z. Liu H. Zhong Z.. (2022). Changes in gut microbiota composition associated with the presence of enteric Protist Blastocystis in captive Forest musk deer (*Moschus Berezovskii*). Microbiol. Spectr. 10:e0226921. doi: 10.1128/spectrum.02269-21, PMID: 35736237 PMC9430526

[ref19] Dermeche S. Nadour M. Larroche C. Moulti-Mati F. Michaud P. (2013). Olive mill wastes: biochemical characterizations and valorization strategies. Process Biochem. 48, 1532–1552. doi: 10.1016/j.procbio.2013.07.010

[ref20] Dworkin M. Falcom S. Rosenberg E. Schleifer K. Stackebrandt E. (2006). The prokaryotes a handbook on the biology of bacteria. New York: Springer.

[ref21] Fernandes A. D. Macklaim J. M. Linn T. G. Reid G. Gloor G. B. (2013). ANOVA-like differential expression (ALDEx) analysis for mixed population RNA-Seq. PLoS One 8:e67019. doi: 10.1371/journal.pone.0067019, PMID: 23843979 PMC3699591

[ref22] Ferrer P. Calvet S. García-Rebollar P. De Blas C. Jiménez-Belenguer A. I. Hernández P.. (2020). Partially defatted olive cake in finishing pig diets: implications on performance, faecal microbiota, carcass quality, slurry composition and gas emission. Animal 14, 426–434. doi: 10.1017/S1751731119002040 31566173

[ref23] Ferrer P. García-Rebollar P. Calvet S. De Blas C. Piquer O. Rodríguez C. A.. (2021). Effects of Orange pulp conservation methods (dehydrated or ensiled Sun-dried) on the nutritional value for finishing pigs and implications on potential gaseous emissions from slurry. Animals 11:387. doi: 10.3390/ani11020387, PMID: 33546423 PMC7913570

[ref24] Ferrer P. García-Rebollar P. Cerisuelo A. Ibáñez M. A. Rodríguez C. A. Calvet S.. (2018). Nutritional value of crude and partially defatted olive cake in finishing pigs and effects on nitrogen balance and gaseous emissions. Anim. Feed Sci. Technol. 236, 131–140. doi: 10.1016/j.anifeedsci.2017.12.014

[ref25] Fouhse J. M. Zijlstra R. T. Willing B. P. (2016). The role of gut microbiota in the health and disease of pigs. Anim. Front. 6, 30–36. doi: 10.2527/af.2016-0031

[ref26] Fuerst J. A. Sagulenko E. (2013). Nuclear and other intracellular compartments in Planctomycetes. J. Mol. Microbiol. Biotechnol. 23, 95–103. doi: 10.1159/000346544, PMID: 23615198

[ref27] Glitso L. V. Brunsgaard G. Hojsgaard S. Sandstrom B. Bach Knudsen K. E. (1998). Intestinal degradation in pigs of rye dietary fibre with different structural characteristics. Br. J. Nutr. 80, 457–468. doi: 10.1017/S0007114598001536, PMID: 9924268

[ref28] Goris T. Cuadrat R. R. C. Braune A. (2021). Flavonoid-modifying capabilities of the human gut microbiome-an *in silico* study. Nutrients 13:2688. doi: 10.3390/nu13082688, PMID: 34444848 PMC8398226

[ref29] Grilli E. Vitari F. Domeneghini C. Palmonari A. Tosi G. Fantinati P.. (2013). Development of a feed additive to reduce caecal *Campylobacter jejuni* in broilers at slaughter age: from *in vitro* to *in vivo*, a proof of concept. J. Appl. Microbiol. 114, 308–317. doi: 10.1111/jam.12053, PMID: 23110383

[ref30] Gupta R. Mahmoud S. Adeolou M. (2013). A phylogenomic and molecular signature based approach for characterization of the phylum Spirochaetes and its major clades: proposal for a taxonomic revision of the phylum. Front. Microbiol. 4:322. doi: 10.3389/fmicb.2013.00217, PMID: 24198815 PMC3814087

[ref31] Jha R. Fouhse J. M. Tiwari U. P. Li L. Willing B. P. (2019). Dietary Fiber and intestinal health of Monogastric animals. Front. Vet. Sci. 6:48. doi: 10.3389/fvets.2019.00048, PMID: 30886850 PMC6409295

[ref32] Jouany J. P. (1982). Volatile fatty acid and alcohol determination in digestive contents silage juices bacterial cultures and anaerobic fermentor contents. Sci. Aliments 4, 171–175. doi: 10.4236/pp.2013.42024

[ref33] Kelly C. J. Zheng L. Campbell E. L. Saeedi B. Scholz C. C. Bayless A. J.. (2015). Crosstalk between microbiota-derived short-chain fatty acids and intestinal epithelial HIF augments tissue barrier function. Cell Host Microbe 17, 662–671. doi: 10.1016/j.chom.2015.03.005, PMID: 25865369 PMC4433427

[ref35] Kim H. B. Borewicz K. White B. A. Singer R. S. Sreevatsan S. Tu Z. J.. (2011). Longitudinal investigation of the age-related bacterial diversity in the feces of commercial pigs. Vet. Microbiol. 153, 124–133. doi: 10.1016/j.vetmic.2011.05.021, PMID: 21658864

[ref36] Klindworth A. Pruesse E. Schweer T. Peplies J. Quast C. Horn M.. (2013). Evaluation of general 16S ribosomal RNA gene PCR primers for classical and next-generation sequencing-based diversity studies. Nucleic Acids Res. 41:e1. doi: 10.1093/nar/gks808, PMID: 22933715 PMC3592464

[ref37] Kong C. Adeola O. (2014). Evaluation of amino acid and energy utilization in feedstuff for swine and poultry diets. Asian Australas. J. Anim. Sci. 27, 917–925. doi: 10.5713/ajas.2014.r.02, PMID: 25050031 PMC4093562

[ref38] Lê S. Josse J. Husson F. (2008). FactoMineR: an R package for multivariate analysis. J. Stat. Softw. 25, 1–18. doi: 10.18637/jss.v025.i01

[ref39] Le Sciellour M. Labussière E. Zemb O. Renaudeau D. (2018). Effect of dietary fiber content on nutrient digestibility and fecal microbiota composition in growing-finishing pigs. PLoS One 13:e0206159. doi: 10.1371/journal.pone.0206159, PMID: 30356293 PMC6200266

[ref40] Li H. Li H. Xie P. Li Z. Yin Y. Blachier F.. (2019). Dietary supplementation with fermented Mao-tai lees beneficially affects gut microbiota structure and function in pigs. AMB Express 9:26. doi: 10.1186/s13568-019-0747-z, PMID: 30778768 PMC6379501

[ref41] Liehr M. Mereu A. Pastor J. J. Quintela J. C. Staats S. Rimbach G.. (2017). Olive oil bioactives protect pigs against experimentally-induced chronic inflammation independently of alterations in gut microbiota. PLoS One 12:e0174239. doi: 10.1371/journal.pone.0174239, PMID: 28346507 PMC5367713

[ref42] Lindberg J. E. (2014). Fiber effects in nutrition and gut health in pigs. J. Anim. Sci. Biotechnol. 5:15. doi: 10.1186/2049-1891-5-15, PMID: 24580966 PMC3975931

[ref43] López-Salas L. Cea I. Borrás-Linares I. Emanuelli T. Robert P. Segura-Carretero A.. (2021). Preliminary investigation of different drying systems to preserve Hydroxytyrosol and its derivatives in olive oil filter cake pressurized liquid extracts. Food Secur. 10:1407. doi: 10.3390/foods10061407, PMID: 34207005 PMC8234471

[ref44] Makkar H. P. S. Ankers P. (2014). Towards sustainable animal diets: a survey-based study. Anim. Feed Sci. Technol. 198, 309–322. doi: 10.1016/j.anifeedsci.2014.09.018

[ref45] Martínez M. Prieto I. Hidalgo M. Segarra A. B. Martínez-Rodríguez A. M. Cobo A.. (2019). Refined versus extra virgin olive oil high-fat diet impact on intestinal microbiota of mice and its relation to different physiological variables. Microorganisms 7:61. doi: 10.3390/microorganisms7020061, PMID: 30813410 PMC6406240

[ref46] Millar M. Barlow A. Williamson S. Higgins R. Stevenson B. (2011). Necrotic ulcerative spirochaetal stomatitis in outdoor pigs. Vet. Rec. 169:55. doi: 10.1136/vr.d4324, PMID: 21742700

[ref47] Molina-Alcaide E. Yáñez-Ruiz D. R. (2008). Potential use of olive by-products in ruminant feeding: a review. Anim. Feed Sci. Technol. 147, 247–264. doi: 10.1016/j.anifeedsci.2007.09.021

[ref48] Molist A. Gómez de Segura A. Pérez J. F. Bhandari S. K. Krause D. O. Nyachoti C. M. (2010). Effect of wheat bran on the health and performance of weaned pigs challenged with *Escherichia coli* K88+. Livest. Sci. 133, 214–217. doi: 10.1016/j.livsci.2010.06.067

[ref49] Moorthy M. Chaiyakunapruk N. Jacob S. A. Palanisamy U. D. (2020). Prebiotic potential of polyphenols, its effect on gut microbiota and anthropometric/clinical markers: a systematic review of randomised controlled trials. Trends Food Sci. Technol. 99, 634–649. doi: 10.1016/j.tifs.2020.03.036

[ref51] Niu Q. Li P. Hao S. Zhang Y. Kim S. W. Li H.. (2015). Dynamic distribution of the gut microbiota and the relationship with apparent crude Fiber digestibility and growth stages in pigs. Sci. Rep. 5:9938. doi: 10.1038/srep09938, PMID: 25898122 PMC4404679

[ref52] Oksanen J. Simpson G. L. Blanchet F. G. Kindt R. Legendre P. Minchin P. R.. (2022). Vegan: Community ecology package. Available at: http://CRAN.Rproject.org/package=vegan.

[ref53] Peterson C. T. Perez Santiago J. Lablokov S. N. Chopra D. Rodionov D. A. Peterson S. N. (2022). Short-chain fatty acids modulate healthy gut microbiota composition and functional potential. Curr. Microbiol. 79:128. doi: 10.1007/s00284-022-02825-5, PMID: 35287182 PMC8921067

[ref54] Piquer L. Calvet S. García-Rebollar P. Cano C. Belloumi D. Cerisuelo A. (2022). “Feeding by-products from the olive industry modifies pig slurry characteristics and ammonia emission” in 73rd annual meeting of EAAP, vol. 50 (Porto), 532.

[ref55] Pollock J. Hutchings M. R. Hutchings K. E. K. Gally D. L. Houdijk J. G. M. (2019). Changes in the ileal, but not fecal, microbiome in response to increased dietary protein level and enterotoxigenic *Escherichia coli* exposure in pigs. Appl. Environ. Microbiol. 85, e01252–e01219. doi: 10.1128/AEM.01252-19, PMID: 31324635 PMC6752020

[ref56] Pu S. Khazanehei H. Jones P. J. Khalipour E. (2016). Interactions between obesity status and dietary intake of monounsaturated and polyunsaturated oils on human gut microbiome profiles in the canola oil multicenter intervention trial (COMIT). Front. Microbiol. 7:1612. doi: 10.3389/fmicb.2016.01612, PMID: 27777570 PMC5056191

[ref57] Pu G. Li P. Du T. Niu Q. Fan L. Wang H.. (2020). Adding appropriate Fiber in diet increases diversity and metabolic capacity of distal gut microbiota without altering Fiber digestibility and growth rate of finishing pig. Front. Microbiol. 11:533. doi: 10.3389/fmicb.2020.00533, PMID: 32328041 PMC7160236

[ref58] R Development Core Team (2012). R: A language and environment for statistical computing. Vienna: R foundation for Statistical Computing Available at: http://www.R-project.org/.

[ref59] Ramayo-Caldas Y. Mach N. Lepage P. Levenez F. Denis C. Lemonnier G.. (2016). Phylogenetic network analysis applied to pig gut microbiota identifies an ecosystem structure linked with growth traits. ISME J. 10, 2973–2977. doi: 10.1038/ismej.2016.77, PMID: 27177190 PMC5148198

[ref60] Rocchetti G. Callegari M. L. Senizza A. Giuberti G. Ruzzolini J. Romani A.. (2022). Oleuropein from olive leaf extracts and extra-virgin olive oil provides distinctive phenolic profiles and modulation of microbiota in the large intestine. Food Chem. 380:132187. doi: 10.1016/j.foodchem.2022.132187, PMID: 35086016

[ref61] Rodríguez-Daza M. C. Pulido-Mateos E. C. Lupien-Meilleur J. Guyonnet D. Desjardins Y. Roy D. (2021). Polyphenol-mediated gut microbiota modulation: toward prebiotics and further. Front. Nutr. 8:689456. doi: 10.3389/fnut.2021.689456, PMID: 34268328 PMC8276758

[ref62] Russo N. Floridia V. D’Alessandro E. Lopreiato V. Pino A. Chiofalo V.. (2023). Influence of olive cake dietary supplementation on fecal microbiota of dairy cows. Front. Microbiol. 14:1137452. doi: 10.3389/fmicb.2023.1137452, PMID: 37206333 PMC10188969

[ref63] Salemdeeb R. Zu Ermgassen E. K. H. J. Kim M. H. Balmford A. Al-Tabbaa A. (2017). Environmental and health impacts of using food waste as animal feed: a comparative analysis of food waste management options. J. Clean. Prod. 140, 871–880. doi: 10.1016/j.jclepro.2016.05.049, PMID: 28050118 PMC5127519

[ref64] Sánchez C. J. Barrero-Domínguez B. Martínez-Miró S. Madrid J. Baños A. Aguinaga M. A.. (2022). Use of olive pulp for gestating Iberian sow feeding: influence on performance, health status indicators, and fecal microbiota. Animals 12:3178. doi: 10.3390/ani12223178, PMID: 36428405 PMC9686466

[ref65] Sato Y. Kuroki Y. Oka K. Takahashi M. Rao S. Sukegawa S.. (2019). Effects of dietary supplementation with *Enterococcus faecium* and *Clostridium butyricum*, either alone or in combination, on growth and fecal microbiota composition of post-weaning pigs at a commercial farm. Front. Vet. Sci. 6:26. doi: 10.3389/fvets.2019.00026, PMID: 30873417 PMC6404372

[ref66] Schmieder R. Edwards R. (2011). Quality control and preprocessing of metagenomic datasets. Bioinformatics 27, 863–864. doi: 10.1093/bioinformatics/btr026, PMID: 21278185 PMC3051327

[ref9001] Standard Methods Committee of the American Public Health Association (2023). American Water Works Association, and Water Environment Federation. 4500-nh3 nitrogen (ammonia) In: Standard Methods For the Examination of Water and Wastewater. 24th Edtn. Lipps WC, Baxter TE,Braun-Howland E, editors. Washington DC: APHA Press. doi: 10.2105/SMWW.2882.087

[ref68] Sun J. Monagas M. Jang S. Molokin A. Harnly J. M. Urban J. F.. (2015). A high fat, high cholesterol diet leads to changes in metabolite patterns in pigs-a metabolomic study. Food Chem. 173, 171–178. doi: 10.1016/j.foodchem.2014.09.161, PMID: 25466009 PMC4255139

[ref69] Tan F. P. Y. Beltranena E. Zijlstra R. T. (2021). Resistant starch: implications of dietary inclusion on gut health and growth in pigs: a review. J. Anim. Sci. Biotechnol. 12:124. doi: 10.1186/s40104-021-00644-5, PMID: 34784962 PMC8597317

[ref70] Tang Q. Shen D. Dai P. Liu J. Zhang M. Deng K.. (2023). Pectin alleviates the pulmonary inflammatory response induced by PM2.5 from a pig house by modulating intestinal microbiota. Ecotoxicol. Environ. Saf. 261:115099. doi: 10.1016/j.ecoenv.2023.115099, PMID: 37285678

[ref71] Trachsel J. M. Bearson B. L. Kerr B. J. Shippy D. C. Byrne K. A. Loving C. L.. (2022). Short chain fatty acids and bacterial taxa associated with reduced *Salmonella enterica* serovar I 4,[5],12:i:- shedding in swine fed a diet supplemented with resistant potato starch. Microbiol. Spectr. 10:e0220221. doi: 10.1128/spectrum.02202-21, PMID: 35532355 PMC9241843

[ref72] Van Soest P. J. Robertson J. B. Lewis B. A. (1991). Methods for dietary fiber, neutral detergent fiber and nonstarch polysaccharides in relation to animal nutrition. J. Dairy Sci. 74, 3583–3597. doi: 10.3168/jds.S0022-0302(91)78551-2, PMID: 1660498

[ref90001] Vasquez R. Oh J. K. Song J. H. Kang D. K. (2022). Gut microbiome-produced metabolites in pigs: a review on their biological functions and the influence of probiotics. J Anim Sci Technol, 64, 671–695. doi: 10.5187/jast.2022.e58, PMID: 35969697 PMC9353353

[ref73] Wang M. Firrman J. Zhang L. Arango-Argoty G. Tomasula P. Liu L.. (2017). Apigenin impacts the growth of the gut microbiota and alters the gene expression of Enterococcus. Molecules 22:1292. doi: 10.3390/molecules22081292, PMID: 28771188 PMC6152273

[ref74] Wang T. Guan K. Su Q. Wang X. Yan Z. Kuang K.. (2022). Change of gut microbiota in PRRSV-resistant pigs and PRRSV-susceptible pigs from Tongcheng pigs and large White pigs crossed population upon PRRSV infection. Animals 12:1504. doi: 10.3390/ani12121504, PMID: 35739841 PMC9219425

[ref75] Wang J. Wang X. Yang T. Wei Z. Banerjee S. Friman V. P.. (2021). Livestock manure type affects microbial community composition and assembly during composting. Front. Microbiol. 12:621126. doi: 10.3389/fmicb.2021.621126, PMID: 33828537 PMC8019744

[ref76] Xin J. Zeng D. Wang H. Sun N. Zhao Y. Dan Y.. (2020). Probiotic *Lactobacillus johnsoniiBS15* promotes growth performance, intestinal immunity, and gut microbiota in piglets. Probiotics Antimicrob. Proteins 12, 184–193. doi: 10.1007/s12602-018-9511-y, PMID: 30617949

[ref77] Yang F. Zhang S. Tian M. Chen J. Chen F. Guan W. (2020). Different sources of high fat diet induces marked changes in gut microbiota of nursery pigs. Front. Microbiol. 11:859. doi: 10.3389/fmicb.2020.00859, PMID: 32457725 PMC7221029

[ref78] Yang P. Zhao J. (2021). Variations on gut health and energy metabolism in pigs and humans by intake of different dietary fibers. Food Sci. Nutr. 9, 4639–4654. doi: 10.1002/fsn3.2421, PMID: 34401110 PMC8358348

[ref79] Zhang L. Jing J. Han L. Wang J. Zhang W. Liu Z.. (2021). Characterization of gut microbiota, metabolism and cytokines in benzene-induced hematopoietic damage. Ecotoxicol. Environ. Saf. 228:112956. doi: 10.1016/j.ecoenv.2021.112956, PMID: 34781132

[ref80] Zhao J. Bai Y. Tao S. Zhang G. Wang J. Liu L.. (2019). Fiber-rich foods affects gut bacterial community and short-chain fatty acids production in pig model. J. Funct. Foods 57, 266–274. doi: 10.1016/j.jff.2019.04.009

[ref81] Zhao J. Bai Y. Zhang G. Liu L. Lai C. (2020). Relationship between dietary Fiber fermentation and volatile fatty acids´ concentration in growing pigs. Animals 10:263. doi: 10.3390/ani10020263, PMID: 32045993 PMC7070776

[ref82] Zhou L. Fang L. Sun Y. Su Y. Zhu W. (2016). Effects of the dietary protein level on the microbial composition and metabolomic profile in the hindgut of the pig. Anaerobe 38, 61–69. doi: 10.1016/j.anaerobe.2015.12.009, PMID: 26723572

