# Supplementary material for: Impact of including two types of destoned olive cakes in pigs’ diets on fecal bacterial composition and study of the relationship between fecal microbiota, feed efficiency, gut fermentation, and gaseous emissions
Source: Front Microbiol. 2024 Jun 12;15:1359670. doi: 10.3389/fmicb.2024.1359670 (PMC11211982; doi:10.3389/fmicb.2024.1359670)
Supplement: Supplementary file 1 [file Table_1.docx]

Supplementary Material

Impact of including two types of destoned olive cakes in pigs’ diets on fecal bacterial composition and study of the relationship between fecal microbiota, feed efficiency, gut fermentation, and gaseous emissions

Dhekra Belloumi^1,2^, Paloma García-Rebollar^3^, Salvador Calvet^2^, M. Pilar Francino^4,5^, Mariana Reyes-Prieto^6^, Jorge González-Garrido^6^, Laia Piquer^1^, Ana Isabel Jiménez-Belenguer^7^, Almudena Bermejo^8^, Carmen Cano^1^, Alba Cerisuelo^1*^

*** Correspondence:** Corresponding Author: [cerisuelo_alb@gva.es](mailto:cerisuelo_alb@gva.es)

Supplementary Table 1. Sample sequences information

| Sample | Total  sequences | Filtered sequences | Merged sequences | No Chimeras sequences |
| --- | --- | --- | --- | --- |
| S01 | 567435 | 55301 | 48499 | 46069 |
| S02 | 505777 | 51690 | 45183 | 43549 |
| S03 | 545312 | 55673 | 49201 | 47504 |
| S04 | 292427 | 29643 | 25712 | 25116 |
| S05 | 777833 | 76716 | 67967 | 65209 |
| S06 | 665720 | 67363 | 59751 | 56500 |
| S07 | 637324 | 64557 | 56232 | 54277 |
| S08 | 449336 | 46211 | 40745 | 38935 |
| S09 | 654857 | 64886 | 57388 | 55231 |
| S10 | 549422 | 54045 | 47953 | 45189 |
| S11 | 562005 | 55797 | 49591 | 47867 |
| S12 | 496912 | 47587 | 42434 | 40567 |
| S13 | 487593 | 48974 | 42615 | 40786 |
| S14 | 288582 | 29489 | 25463 | 24695 |
| S16 | 175370 | 18010 | 15180 | 14749 |
| S17 | 335546 | 33194 | 29256 | 28458 |
| S18 | 409435 | 41572 | 36516 | 35526 |
| S19 | 324108 | 33397 | 29438 | 28547 |
| S20 | 282548 | 29275 | 25625 | 25062 |
| S21 | 381819 | 38152 | 33577 | 32291 |
| S22 | 387868 | 38567 | 33195 | 32077 |
| S23 | 96428 | 9214 | 7686 | 7372 |
| S24 | 370117 | 37100 | 32550 | 31158 |
| S25 | 462900 | 46516 | 41384 | 39530 |
| S26 | 262992 | 27409 | 23906 | 23239 |
| S27 | 375981 | 39053 | 34984 | 34012 |
| S28 | 311025 | 32531 | 28589 | 27784 |
| S29 | 297691 | 29563 | 26232 | 25418 |
| S30 | 330439 | 33789 | 29423 | 28780 |
| S31 | 358347 | 36906 | 32703 | 31792 |
| S32 | 279337 | 29459 | 25585 | 24763 |
| S33 | 371473 | 37599 | 33714 | 32207 |
| S34 | 464285 | 47241 | 41399 | 39919 |
| S35 | 339126 | 34835 | 30083 | 28987 |
| S36 | 426485 | 41323 | 37342 | 36118 |
| S37 | 454321 | 43915 | 38406 | 36710 |
| S38 | 303029 | 29439 | 25443 | 24312 |
| S39 | 15624 | 1110 | 800 | 786 |
| S40 | 315422 | 30866 | 26913 | 26111 |
| S41 | 314762 | 28371 | 24574 | 23659 |
| S42 | 338493 | 32370 | 28304 | 27682 |
| S43 | 348652 | 33596 | 30085 | 29059 |
| S44 | 293064 | 29006 | 25453 | 24530 |
| S45 | 381441 | 36156 | 31459 | 30442 |
| S46 | 451144 | 42375 | 37243 | 36410 |
| S47 | 229611 | 19417 | 16695 | 16002 |
| S48 | 425547 | 40841 | 36539 | 35467 |
| S49 | 461374 | 45410 | 40544 | 39293 |
| S50 | 247677 | 25202 | 22284 | 21624 |
| S51 | 359702 | 37204 | 33483 | 31958 |
| S52 | 382749 | 39936 | 35428 | 33540 |
| S53 | 332619 | 32026 | 28272 | 27378 |
| S54 | 323811 | 32304 | 29005 | 28333 |
| S55 | 358819 | 35886 | 31667 | 30794 |
| S56 | 322065 | 32278 | 28604 | 27839 |
| S57 | 315468 | 30280 | 26168 | 25408 |
| S58 | 425746 | 42471 | 37749 | 36576 |
| S59 | 423612 | 42154 | 37593 | 36138 |
| S60 | 387122 | 35725 | 30795 | 29976 |
| S61 | 453395 | 45677 | 39776 | 37945 |
